# The transcriptional landscape of plant infection by the rice blast fungus *Magnaporthe oryzae* reveals distinct families of temporally co-regulated and structurally conserved effectors

**DOI:** 10.1093/plcell/koad036

**Published:** 2023-02-18

**Authors:** Xia Yan, Bozeng Tang, Lauren S Ryder, Dan MacLean, Vincent M Were, Alice Bisola Eseola, Neftaly Cruz-Mireles, Weibin Ma, Andrew J Foster, Miriam Osés-Ruiz, Nicholas J Talbot

**Affiliations:** The Sainsbury Laboratory, University of East Anglia, Norwich Research Park, Norwich NR4 7UH, UK; The Sainsbury Laboratory, University of East Anglia, Norwich Research Park, Norwich NR4 7UH, UK; The Sainsbury Laboratory, University of East Anglia, Norwich Research Park, Norwich NR4 7UH, UK; The Sainsbury Laboratory, University of East Anglia, Norwich Research Park, Norwich NR4 7UH, UK; The Sainsbury Laboratory, University of East Anglia, Norwich Research Park, Norwich NR4 7UH, UK; The Sainsbury Laboratory, University of East Anglia, Norwich Research Park, Norwich NR4 7UH, UK; The Sainsbury Laboratory, University of East Anglia, Norwich Research Park, Norwich NR4 7UH, UK; The Sainsbury Laboratory, University of East Anglia, Norwich Research Park, Norwich NR4 7UH, UK; The Sainsbury Laboratory, University of East Anglia, Norwich Research Park, Norwich NR4 7UH, UK; The Sainsbury Laboratory, University of East Anglia, Norwich Research Park, Norwich NR4 7UH, UK; The Sainsbury Laboratory, University of East Anglia, Norwich Research Park, Norwich NR4 7UH, UK

## Abstract

The rice blast fungus *Magnaporthe oryzae* causes a devastating disease that threatens global rice (*Oryza sativa*) production. Despite intense study, the biology of plant tissue invasion during blast disease remains poorly understood. Here we report a high-resolution transcriptional profiling study of the entire plant-associated development of the blast fungus. Our analysis revealed major temporal changes in fungal gene expression during plant infection. Pathogen gene expression could be classified into 10 modules of temporally co-expressed genes, providing evidence for the induction of pronounced shifts in primary and secondary metabolism, cell signaling, and transcriptional regulation. A set of 863 genes encoding secreted proteins are differentially expressed at specific stages of infection, and 546 genes named *MEP* (*Magnaporthe*effector protein) genes were predicted to encode effectors. Computational prediction of structurally related *MEPs*, including the MAX effector family, revealed their temporal co-regulation in the same co-expression modules. We characterized 32 *MEP* genes and demonstrate that Mep effectors are predominantly targeted to the cytoplasm of rice cells via the biotrophic interfacial complex and use a common unconventional secretory pathway. Taken together, our study reveals major changes in gene expression associated with blast disease and identifies a diverse repertoire of effectors critical for successful infection.

## Teaser

Transcriptional profiling reveals how an extensive effector repertoire is deployed during rice blast disease, including temporally co-regulated, sequence-unrelated, structurally conserved effectors.

**IN A NUTSHELL**

**Background:** The rice blast fungus *Magnaporthe oryzae* causes a devastating disease that threatens global rice (*Oryza sativa*) production, destroying enough rice each year to feed more than 60 million people. Although the disease has been extensively studied, we still do not understand how the fungus is able to invade rice tissue so effectively and how it overwhelms the plant's defenses.

**Question:** We set out to identify the major changes in gene expression that occur during rice blast disease and to use this information to define the repertoire of fungal effector proteins that are deployed by the fungus during plant infection.

**Findings:** We discovered that the blast fungus has a much more extensive repertoire of effectors than previously thought and these effectors are expressed at specific times during infection. This mirrors the expression of very large sets of fungal genes encoding proteins associated with secondary metabolism, transporter functions, and cell signaling during each stage of pathogenesis. We found that sequence-unrelated but structurally conserved effectors are expressed in a coordinated manner during infection. Many of these effectors are delivered into plant cells, and we developed a sensitive relative fitness assay to show that even an individual effector can contribute to fungal virulence.

**Next steps:** Two main questions emerge from our study. First, what do all of these effectors do? What are their targets and how do they enable the fungus to colonize rice cells so rapidly? Second, how are effector genes temporally and spatially regulated? What are the transcriptional regulators that ensure they are expressed in such specific patterns?

## Introduction

Rice blast disease is one of the most significant factors constraining rice (*Oryza sativa*) production worldwide. Each year, despite the deployment of rice varieties carrying numerous resistance genes and the extensive use of fungicides, between 10% and 30% of the rice harvest is lost to rice blast disease (Yamaguchi 2004; Wang and Valent 2017; Stam and McDonald 2018; Poloni et al. 2020). Given that rice provides 23% of the calories to humankind and is a staple food for half of the world's population, controlling rice blast in a sustainable manner would constitute a major contribution to global food security. Rice blast is caused by the filamentous fungus *Magnaporthe oryzae* (synonym of *Pyricularia oryzae*) (Zhang et al. 2016) which has evolved the ability to breach the tough outer cuticle of rice leaves and invade living plant tissue. *M. oryzae* can also infect a wide range of grass hosts and cause diseases, such as wheat blast, an emerging threat to wheat (*Triticum aestivum*) production (Urashima et al. 1993; Islam et al. 2016; Inoue et al. 2017; Latorre et al. 2022).

The blast fungus undergoes a series of morphogenetic transitions during plant infection, including cellular differentiation to develop infection cells called appressoria on the leaf surface, followed by penetration and rapid proliferation of invasive hyphae within rice cells, a process facilitated by the active suppression of plant immunity. Like many of the most important crop pathogens, the rice blast fungus is a hemibiotroph, which means that it actively grows in living plant tissue during its initial biotrophic phase of development, but later causes plant cell death when it develops necrotrophically, utilizing nutrients released from dead plant cells to enable the fungus to sporulate from necrotic disease lesions.

So far, more than 1,600 *M. oryzae* genes have been functionally characterized by targeted gene deletion or mutagenesis, representing greater than 10% of the rice blast genome (Foster et al. 2021). These studies have provided insight into the biology of blast disease, especially the ability of the fungus to form appressoria on the leaf surface. However, there are many aspects of the biology of blast disease that are not well understood, even after such intense study. We do not know, for example, how the blast fungus is able to invade living plant tissue so rapidly, overcoming host defenses, colonizing new plant cells, and spreading long distances throughout rice leaves. To be such an efficient invader of plant cells, *M. oryzae* must be able to adapt successfully to the host environment—sequestering carbon and nitrogen sources to fuel its growth—while evading recognition by the host, but how this is achieved is not clear. The blast fungus also induces profound changes in the organization of plant cells, including extensive membrane biogenesis, changes in cytoskeletal configuration, and perturbation of cell-to-cell communication. How these processes are orchestrated and regulated by the invading fungus also remains largely unknown. One of the principal reasons for our current lack of understanding is that few investigations have taken a holistic view of plant infection—in contrast to gene functional studies—attempting to understand the progression of blast disease and the major temporal changes in pathogen physiology.

In this study, we set out to define the transcriptional landscape of rice blast infection. Our aim was to identify major changes in pathogen gene expression from the moment of initial inoculation of plants until the development of disease symptoms and, in particular, to use this information to identify the full repertoire of fungal effector proteins deployed by the fungus.

Effectors are secreted proteins that target components of the plant immune system to suppress host defense and enable proliferation of the pathogen (Jones and Dangl 2006; Kamoun 2006; Lo Presti et al. 2015). In addition to suppressing plant immunity, effectors may target cell signaling and metabolic processes to facilitate invasive fungal growth (Zhai et al. 2022). In *M. oryzae,* effectors target extracellular processes such as chitin-triggered immunity that operate in the apoplast (Mentlak et al. 2012), or intracellular processes such as the perturbation of reactive oxygen species generation (Liu and Zhang 2021), targeted protein degradation (Park et al. 2012), or reprogramming the host transcription (Kim et al. 2020). Intracellular effectors accumulate in a membrane-rich plant structure called the biotrophic interfacial complex (BIC), which appears necessary for their delivery into plant cells (Kankanala et al. 2007). Four cytoplasmic effectors have been shown to be secreted by an unusual Golgi-independent mechanism (Giraldo et al. 2013). A sub-set of *M. oryzae* effectors are recognized by rice immune receptors, leading to disease resistance. The interaction of such avirulence effectors (Avrs) with cognate nucleotide-binding leucine-rich repeat receptors (NLRs) has also helped reveal their likely intracellular targets, especially in cases where specific effector-binding domains have become integrated into NLRs (Cesari et al. 2013; Maqbool et al. 2015; De la Concepcion et al. 2018). Several sequence non-related Avr effectors possess a conserved protein fold and are termed MAX (Magnaporthe Avrs and ToxB like) effectors (de Guillen et al. 2015). Structural modeling of the predicted secreted proteome of *M. oryzae* has recently identified large sets of structurally related proteins (Seong and Krasileva 2021b), although their roles in blast disease are not yet known.

We reasoned that comprehensive transcriptional profiling would provide a means to systematically analyze the landscape of blast disease at a holistic level and reveal the true effector repertoire of *M. oryzae*. Transcriptomic studies have, for instance, provided key insights into physiologically complex states, such as tumorigenesis (Wingrove et al. 2019), embryonic development (He et al. 2020), and host immunity (Bjornson et al. 2021), as well as fungal–plant interactions (Copley et al. 2017; Zeng et al. 2017; Lanver et al. 2018). Previous attempts to define global patterns of gene expression in the *M. oryzae*–rice interaction have utilized methods such as microarrays and super-SAGE analysis or, more recently, by RNA-seq (Mosquera et al. 2009; Kawahara et al. 2012; Soanes et al. 2012; Chen et al. 2013; Dong et al. 2015; Sharpee et al. 2017; Shimizu et al. 2019). These studies have suffered from the lower resolution of previous methodologies, failing to detect gene expression from the low fungal biomass during early stages of plant infection. Relatively poor coverage of fungal gene expression changes has been reported, with only the most abundantly expressed fungal genes identified. More recent studies using RNA-seq analysis have benefited from the dynamic range and resolution of deep sequencing, but these investigations have focused (almost exclusively) on a single time point during infection, leading to assumptions regarding the progress of disease that have severe limitations. Moreover, most transcriptomic studies of *M. oryzae* have generated only superficial coverage of fungal gene expression changes due to the type of inoculation methods carried out and the relatively poor rates of blast infection observed.

In this project, we set out to overcome the major limitations of these studies by carrying out a comprehensive time-course of rice blast disease using different infection protocols and rice hosts to enhance the efficiency of blast infection. We reasoned that by infecting rice cultivars of varying blast susceptibility, coupled with using different inoculation methods, we could optimize the number of fungal genes analyzed and thereby generate deeper insights into the transcriptional landscape of plant infection. We performed RNA-seq analysis of *M. oryzae* strain Guy11 infecting 2 rice cultivars with different levels of susceptibility, either by spray infections or using leaf drop inoculation of attached rice leaves. We report the global pattern of fungal gene expression at 8 time points from 0 h (the time of spore inoculation) until full blast symptom expression at 144 h. In this way, we were able to define 10 modules of temporally co-expressed fungal genes and define physiological, metabolic, and gene regulatory networks represented by each module. We identified 863 secreted protein-encoding genes that are differentially regulated during plant infection, many of which are predicted to encode effectors. The effector repertoire of *M. oryzae* and its temporal expression dynamics are, therefore, much greater in complexity than previously recognized. Strikingly, we also found that effector candidates predicted to be structurally conserved (Seong and Krasileva 2021a, 2021b) are temporally co-expressed during biotrophic growth. Using live-cell imaging and gene functional analysis, we report the cell biological features of these effectors and their specific positioning during infection. When considered together, our findings provide important insights into the biology of blast disease and the complexity of the deployed effector repertoire.

## Results

To examine the transcriptional landscape of rice blast disease, we selected 2 rice cultivars differing in their susceptibility to blast. CO39 is a dwarf *indica* rice variety with moderate susceptibility to blast that has been used as a host in many gene functional studies of *M. oryzae* (Talbot et al. 1993a; Chauhan et al. 2002; Zhu et al. 2004). Moukoto is a *japonica* rice variety that is highly susceptible to blast disease, resulting in large, coalescing disease lesions (Yoshida et al. 2009). We inoculated 21-d-old rice seedlings by spray infection with 1 × 10^5^ conidia mL^−1^, as well as carrying out leaf drop infections in which a 20-μl drop of a suspension of 1 × 10^6^ conidia mL^−1^ was placed on the surface of a rice leaf that remained attached to a 21-d-old rice seedling. The compatible *M. oryzae* strain Guy11 was used for all infections.

**Figure 1.**
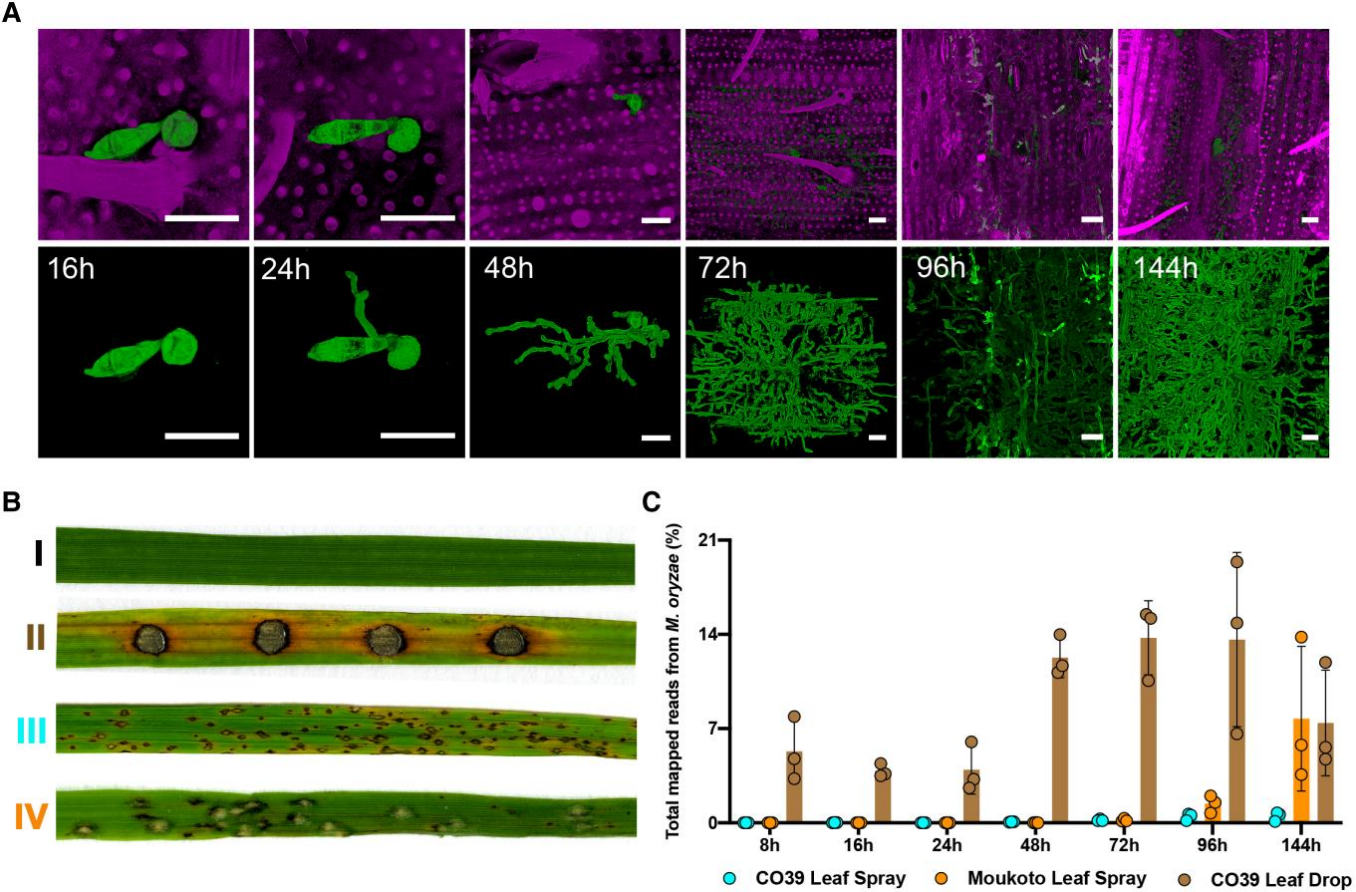
Transcriptional profile analysis of a time-course of plant infection by the rice blast fungus *M. oryzae.* Rice infections were carried out using 2 distinct inoculation methods and 2 cultivars differing in relative susceptibility to blast. **A)** Micrographs of rice cultivar CO39 leaves inoculated with *M. oryzae* Guy11 to show the progression of tissue invasion. Infected rice leaves were collected at 16-, 24-, 48-, 72-, 96-, and 144-h postinoculation. Wheat Germ Agglutinin-Alexa Fluor 488 conjugate (WGA-AF488) was used to stain fungal hyphae and PI was used to stain the plant cell wall. Scale bars = 20 µm. **B)** Comparison of rice blast disease symptoms 6-d postinoculation using either leaf drop infection or spray infection on rice cultivars with varying host susceptibility. I: moderately susceptible rice cultivar CO39 inoculated with water control; II: leaf drop infection of rice CO39 with Guy11; III: spray infection of rice CO39 with Guy11; IV: spray infection of highly susceptible rice cultivar Moukoto with Guy11. **C)** Graph depicting the proportion of fungal transcripts in the plant and pathogen mixed transcriptome (CO39 Leaf Spray, Moukoto Leaf Spray, and CO39 Leaf Drop correspond to inoculation methods). Error bars represent SD of 3 biological replicates for all time points except conidia with 2 biological replicates.

**Figure 2.**
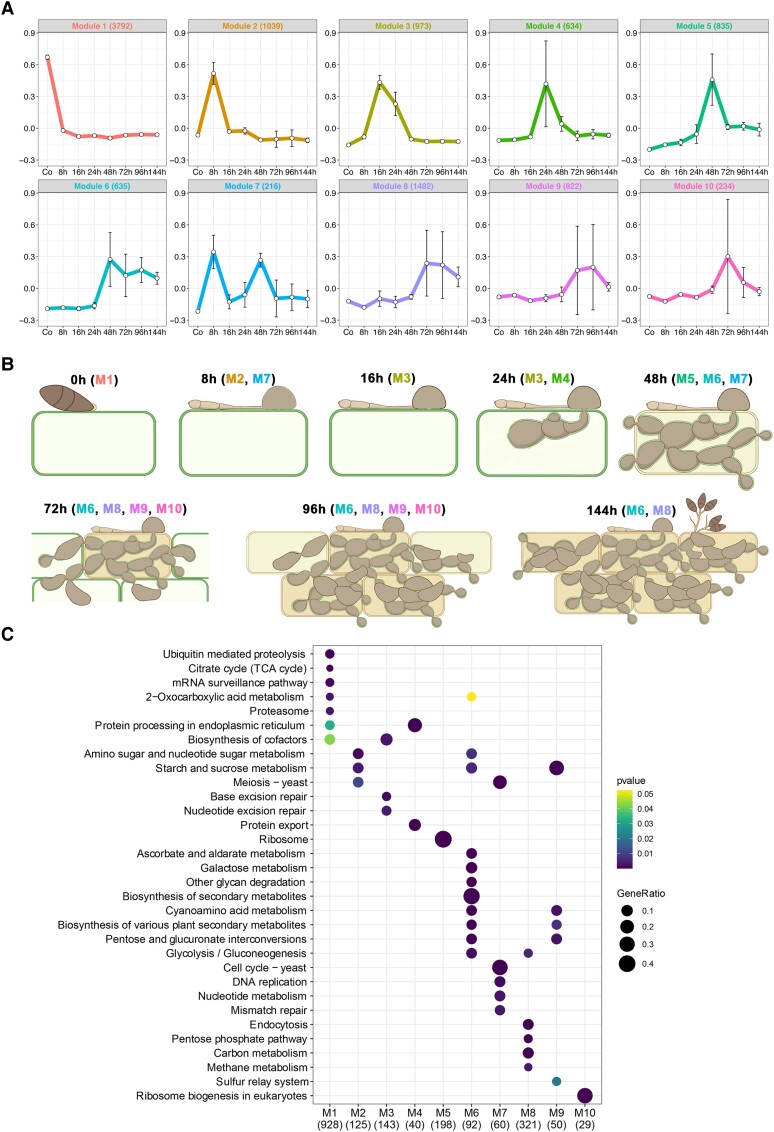
Temporal co-expression analysis reveals 10 modules of pathogen gene expression during rice blast infection. Analysis of co-expressed pathogen genes during rice blast disease development. **A)** Weighted correlation network analysis (WGCNA) identifies 10 co-expressed modules during a time-course of infection-related development and plant infection (Modules 1–10). The representative eigengene is shown for each module. Co, 0-h conidia control. Error bars represent SD of 3 biological replicates for all time points except conidia with 2 biological replicates. **B)** Schematic representation of each stage of rice blast disease development when genes in color-coded corresponding WGCNA modules are co-expressed. **C)** KEGG enrichment analysis of genes in each WGCNA module using clusterProfiler reveals over-represented physiological functions during blast disease development.

The rapid invasion of new rice cells could be observed by staining the fungus with Wheat Germ Agglutinin-Alexa Fluor 488 conjugate (WGA-AF488) and rice tissue with propidium iodide (PI), as shown in Fig. 1A and Supplemental Movies 1–6. All infection experiments were repeated 3 times (3 biological replicates) with *M. oryzae* cultures and rice seedlings of the same age and always inoculated at the same time of day to control for circadian effects (Fig. 1B). The inoculated leaf area was collected for sample extraction at 8 different time points that are key stages for disease development: 0 (uninfected), 8-, 16-, 24-, 48-, 72-, 96-, and 144-h postinfection, as depicted in Fig. 2. These time points cover all morphogenetic transitions associated with appressorium development, including appressorium-mediated penetration, biotrophic growth, transpressorium-dependent cell-to-cell movement, fungal proliferation in rice tissue, the switch to necrotrophic growth, and fungal sporulation (Cruz-Mireles et al. 2021). Using Illumina sequencing of mRNA libraries extracted from rice tissue, we generated 4.37 billion reads from all samples (Supplemental Data Set S1).

Kraken 2 was used to identify *M. oryzae* and rice reads from the mixed transcriptome (Wood et al. 2019). We classified reads either as originating from rice or the rice blast fungus and focus here on reads that mapped to the annotated *M. oryzae* reference genome (Dean et al. 2005). By utilizing the highly susceptible cultivar Moukoto, we were able to show a greater number of *M. oryzae* sequence reads, particularly at later stages of disease progression compared with CO39 infections, as shown in Fig. 1C. However, leaf drop infections were significantly enriched in fungal reads in the mixed transcriptome analysis. The greater number of fungal reads probably reflects a higher proportion of rice cells infected by the fungus in leaf drop samples compared with spray inoculations, so the transcriptome data are likely to be more representative of the infected state. By contrast, in spray inoculations, many rice cells remain uninfected throughout the process and will only respond at a distance from the site of infection (Fig. 1C and Supplemental Fig. S1A). During infection, we observed a 3-fold increase in the proportion of *M. oryzae* reads between 24 and 48 hpi in leaf drop infections (Supplemental Data Set S1), which is consistent with the steep increase in fungal biomass that accompanies invasion of neighboring cells during this 24-h period (Fig. 1, A–C). Principal Component Analysis (PCA) using transcript per kilobase million (TPM) highlighted the reproducibility between biological replicates of each sample (Supplemental Fig. S1B) and also provided evidence of a significant change in gene expression between early stages (8 to 24 hpi) of infection and later stages (48 to 144 hpi), which occurred irrespective of inoculation method (Supplemental Fig. S2A).

### Temporal dynamics of gene expression during rice blast disease

To define the temporal sequence of changes in fungal gene expression during rice infection, we analyzed gene expression across the time-course using weighted gene co-expression network analysis (WGCNA) (Zhang and Horvath 2005; Langfelder and Horvath 2008). This analysis identified 10 modules of co-expressed genes based on the calculation of a correlation coefficient, for which the expression pattern of a representative eigengene is shown in Fig. 2 (see also Supplemental Data Set S2). Modules ranged in size from 216 to 3,792 temporally co-expressed genes (Fig. 2A and Supplemental Data Set S2). Genes in color-coded modules are highly expressed at the developmental stages as shown in Fig. 2B, which is a schematic representation of the infection dynamics during droplet infection of rice leaves (Fig. 1A). Module (M) 1, for example, contains 3,792 genes that show peak expression in conidia and are then downregulated following appressorium-mediated penetration. These include genes involved in fungal growth, conidiation, and spore germination, such as *LEU1* (Tang et al. 2019; Que et al. 2020), *SOM1* (Yan et al. 2011), and *YPT7* (Liu et al. 2015), all previously reported to be necessary for sporulation, as well as genes implicated in appressorium morphogenesis, such as the osmosensor-encoding gene *SHO1* (Liu et al. 2011), the appressorium turgor sensor kinase gene *SLN1* (Zhang et al. 2010; Ryder et al. 2019), and the *RGS* family of genes that regulate G-protein signaling (Zhang et al. 2011). The septin genes were also predominantly classified in M1 (*SEP3*, *SEP4*, and *SEP6*) and M2 (*SEP5*), which is consistent with their vital role in appressorium re-polarization (Dagdas et al. 2012).

Genes associated with appressorium-mediated infection were predominantly grouped within M2, which shows peak expression at 8 hpi (Fig. 2B) and contains 1,039 genes highly expressed during the pre-penetration phase of fungal growth. These include *PTH11* (Sweigard et al. 1998), *BUF1* (Valent et al. 1991), *MAGB* (Liu and Dean 1997), and *ZNF1* (Cao et al. 2016; Yue et al. 2016), all necessary for appressorium development and function. Initial biotrophic colonization of rice tissue is represented by genes in M3, M4, and M5, which show peaks of expression at 16, 24, and 48 hpi, respectively. M3 contains *MSB2* (Liu et al. 2011), *HOX7* (Kim et al. 2009; Oses-Ruiz et al. 2021), and *MET13* (Yan et al. 2013), which are implicated in appressorium function, and *SEC6*, which encodes a component of the octameric exocyst complex that assembles in a septin-dependent manner during plant infection (Gupta et al. 2015). M4 consists of 634 genes, including genes with functions expected during early biotrophic growth, such as *RBF1*, which encodes a protein required for BIC formation (Nishimura et al. 2016), as well as many effectors (see below). M5 contains 835 genes associated with invasive growth, such as the NADPH oxidase subunit-encoding gene *NOXD* (Galhano et al. 2017), as well as the MAPK gene *MPS1* (Xu et al. 1998), *SSB1*, *SSZ1*, and *ZUO1*, which are all involved in the cell wall integrity pathway (Yang et al. 2018). These results are consistent with the substantial change in cell wall organization occurring during invasive growth.

M6 contains genes upregulated in association with the increase in fungal biomass that occurs during invasive growth. This module is also enriched in genes encoding transcription factors and signaling proteins (Supplemental Data Set S2), which are predominantly uncharacterized, but may reflect the dramatic reprogramming in fungal physiology that occurs at the switch from biotrophic growth to necrotrophic growth. M7 shows genes that peak in expression during appressorium development at 8 h and then again during transpressorium development at 48 h. This observation is consistent with the developmental conservation of these 2 infection structures, as recently highlighted (Cruz-Mireles et al. 2021). M7 contains genes such as *MST12*, which encodes a transcription factor required for appressorium-mediated penetration (Park et al. 2002); *CPKA*, encoding the catalytic subunit of cAMP-dependent protein kinase A, another key regulator of appressorium development (Mitchell and Dean 1995); as well as the cell cycle regulator *NIM1* (Saunders et al. 2010) and the peroxin-encoding gene *PEX1* (Deng et al. 2016). Their bi-modal expression profile in M7 is consistent with these functions being implicated in transpressorium morphogenesis. Finally, the switch to necrotrophic growth by *M. oryzae* is represented by M8, M9, and M10, which peak in expression after 72 hpi (Fig. 2B). These modules include genes whose functions are associated with conidiogenesis, such as *SMO1*, encoding a Ras GTPase-Activating Protein, the fatty acid synthase (FAS) beta subunit dehydratase gene *FAS1* (Sangappillai and Nadarajah 2020) and the necrosis- and ethylene-inducing protein 1-encoding gene *NLP1* (Park et al. 2006; Fang et al. 2017; Kershaw et al. 2019).

### Invasive growth of *M. oryzae* involves specific physiological transitions

To identify physiological processes within each WGCNA module of co-expressed genes, we carried out metabolic pathway enrichment analysis, as shown in Fig. 2C. As the temporal progression of rice blast infection proceeds, several waves of gene expression could be identified. Over-representation of genes associated with regulated proteolysis, for example, is a feature of M1, consistent with the role of autophagy in appressorium maturation. There is also a switch from the tricarboxylic acid cycle (M1) to sucrose metabolism (M2, M6, M9) and the pentose phosphate pathway during invasive growth (M8). This is consistent with the Nut1/Pas1/Asd4-regulated NADPH-dependent metabolic regulation previously reported in *M. oryzae* (Wilson et al. 2010) and the metabolic transitions that occur during invasive fungal growth (Fernandez et al. 2014). The physiological signature of biotrophy also includes evidence of rapid fungal proliferation, as exhibited by cell division-associated functions in M2 and M3, as well as protein processing and export in M4 and ribosomal biogenesis in M5. There is also a very pronounced switch that is clear in M6 associated with the appearance of disease symptoms, which occurs at 72 h, and the onset of necrotrophy. M6 shows over-representation of secondary metabolism-associated gene expression and plant cell wall degrading enzymes, for instance. The parallels between appressorium morphogenesis and transpressorium function are also evident by the physiological functions over-represented in M7, including cell cycle control—a key regulator of appressorium development (Saunders et al. 2010)—and associated DNA replication functions. Further secondary metabolic functions are represented in M8 and M9 during the onset of disease symptom development and conidiogenesis.

In a complementary analysis to WGCNA clustering, we defined the expression profiles of all *M. oryzae* genes at each stage during infection. Fungal read counts were normalized and compared to expression in conidial mRNA using Sleuth (Pimentel et al. 2017), thereby identifying temporal changes in gene expression (log2 fold change >1, and *P*-adj < 0.05) that occur during pathogenesis compared with the original inoculum. This analysis revealed that the largest change in *M. oryzae* gene expression occurs at 48 hpi compared with the conidial transcriptome, highlighting the substantial physiological transition associated with invasive fungal growth, with 1,920 genes upregulated and 1,012 downregulated at this time point. Metabolic pathway enrichment analysis revealed the expression of many growth-related functions, including cell division, translation, and regulated proteolysis, as well as the switch to carbohydrate metabolism that accompanies invasive fungal growth (Supplemental Fig. S3 and Supplemental Data Set S2). The identification of differentially expressed genes that showed repression (or marked downregulation) during each stage of pathogenic development provided evidence of a general repression of transport-associated functions and lipid and fatty acid metabolism during invasive growth compared with conidia (Supplemental Fig. S3). This highlights how pathogenic development requires not only the upregulation of specific gene functions, but also the orchestrated repression of many gene functions compared with the relative pluripotency of the germinating spore.

Targeted analysis of secondary metabolic functions revealed the specific expression of genes encoding polyketide synthase (PKS), FAS, and cytochrome P450 mono-oxygenases during plant infection (Supplemental Figs. S4–S6). Conidial-specific expression of 8 PKS genes occurs prior to infection, followed by the co-regulation of 4 PKS genes at 8 h, 6 at 24 h, and 7 at 48 h, which is consistent with the production of specific metabolites during each stage of development (Supplemental Fig. S4). This is mirrored by the differential FAS gene expression occurring during both the biotrophic and necrotrophic stages of development (Supplemental Fig. S5) and, strikingly, by the temporal expression of cytochrome P450 genes at each time point, with 21 genes expressed (for example) specifically at 24 h (Supplemental Fig. S6). Genes encoding key transporters are also highly upregulated at 8 hpi, including *NGT1* in Module 2, which is required for the transport of *N*-acetyl glucosamine (Kumar et al. 2016). Genes encoding ABC transporters responsible for transporting a wide range of substrates are upregulated during plant infection, including *ABC5*, *ABC6*, and *ABC7* (Kim et al. 2013) (Supplemental Fig. S7).

Consistent with these temporal changes in gene expression, a targeted analysis of the 495 predicted transcription factor-encoding genes from the *M. oryzae* genome (Park et al. 2013) showed a pattern of co-expression, with large clusters of co-expressed transcription factor-encoding genes in conidia, particularly at the 8-, 16-, 24-, and 48-h time points (Supplemental Fig. S8). A clear transition in transcriptional regulation occurs after 48 h, with most transcription factor-encoding genes from the earlier biotrophic phase of growth being repressed after this time, accompanied by the expression of a completely separate group of regulators between 72 and 144 h during disease symptom development. This is consistent with a recent report suggesting that histone modification dynamics at H3K27 play an important regulatory role during these stages of invasive growth of *M. oryzae* (Zhang et al. 2021). We conclude that major regulatory changes in gene expression, associated with switches in both primary and secondary metabolism, occur during plant infection and the transition from biotrophic proliferation to necrotrophic growth.

### A large family of *M. oryzae* effector genes is differentially expressed during rice infection

**Figure 3.**
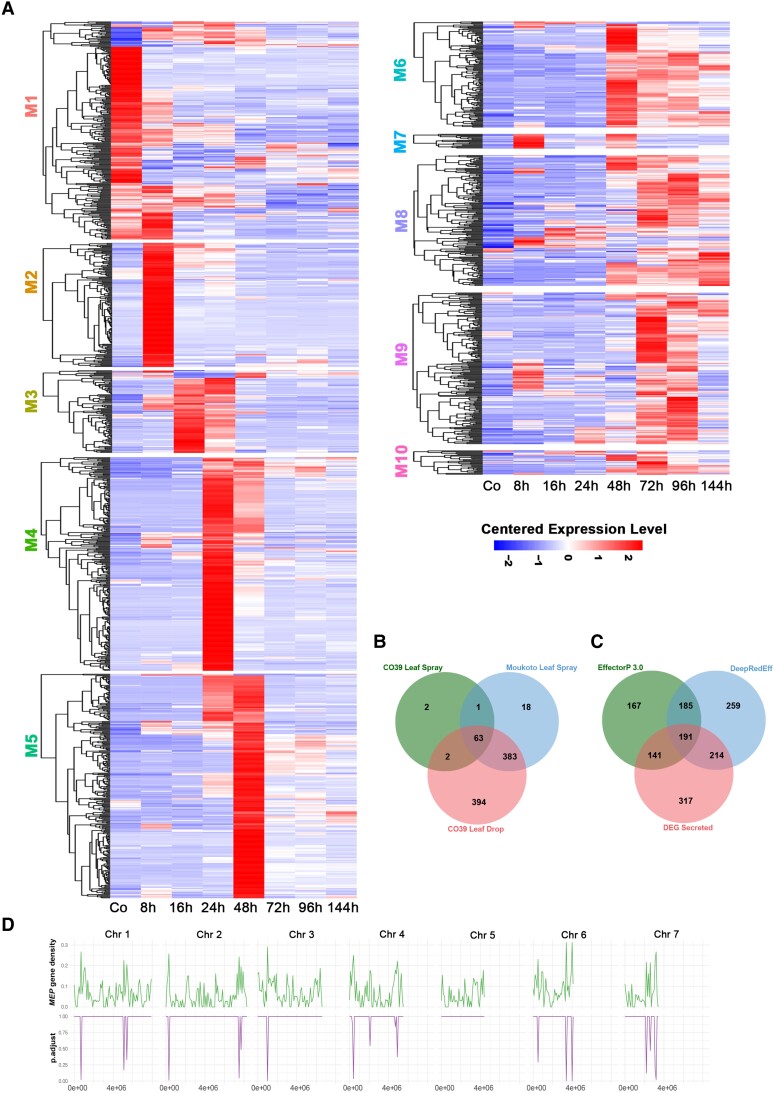
Stage-specific temporal expression of the *M. oryzae* secretome during rice infection. **A)** Heat map showing the hierarchical clustering of pathogen genes encoding putatively secreted proteins from each WGCNA co-expression module. Distinct temporal patterns of secretome expression occur during biotrophic and necrotrophic development. Expression levels are shown relative to the mean expression TPM value across all stages. **B)** Venn diagram showing a comparison of differentially expressed secreted protein-encoding genes from 3 different inoculation experiments (CO39 Leaf Spray, Moukoto Leaf Spray, and CO39 Leaf Drop Infections). In total, 68 effector candidates were identified from the CO39 leaf spray infections, 467 effector candidates from Moukoto leaf spray inoculation, and 847 effector candidates from CO39 leaf drop infections. Although leaf drop-CO39 infections revealed expression of many more differentially expressed effector candidates, 20 effector candidates were revealed only in spray inoculation experiments. **C)** Comparison of effector predictions of host-induced secreted proteins using EffectorP 3.0 and DeepRedEff algorithms with differentially expressed genes encoding signal peptide-containing proteins (DEG Secreted). **D)** Enrichment analysis of *MEP* gene loci on *M. oryzae* chromosomes 1–7, encoding predicted secreted proteins. The number of *MEP* genes was compared with the total gene number in 200 kb windows across each chromosome. Phyper was used to compute the hypergeometric distribution and obtain *P*-values (Johnson et al. 1992). The resulting graphs in the bottom panel show regions of each chromosome where there is a significant over-representation of *MEP* gene loci.

We next focused on defining the expression of *M. oryzae* genes predicted to encode secreted proteins, including potential effector candidates. Secreted proteins were predicted using SignalP from data sets of each inoculation method and strain–cultivar interaction. This revealed 68 candidate effector genes in the samples spray inoculated with CO39, 467 effector candidates in the samples spray inoculated with Moukoto, and 847 putative effectors in samples following leaf drop infection with CO39 (Fig. 3 and Supplemental Data Sets S3–S5); these results are consistent with the increased resolution obtained by using a highly susceptible rice cultivar and leaf drop infection. We then investigated the temporal expression profiles of predicted effector genes and classified them into each WGCNA module (Fig. 3A). This revealed distinct patterns of gene expression, particularly during the initial stages of gene expression (M1–M5), with particular over-representation of effector candidates in M4 and M5. The expression of well-characterized effector-encoding genes corresponded to each module. M3, for example, includes *NIS1*, encoding an effector that suppresses Bak1/Bik1-dependent PAMP-triggered immunity (Mentlak et al. 2012; Irieda et al. 2019); *SLP1*, encoding an apoplastic effector that suppresses chitin-triggered immunity (Mentlak et al. 2012); and *MoSVP1*, encoding an effector required for appressorium-mediated penetration (Shimizu et al. 2019). M4 includes *ACE1*, encoding an effector whose biosynthetic activity is necessary for avirulence to Pi33 (Bohnert et al. 2004); *HEG13*, which antagonizes cell death induced by Nep1 in *Nicotiana benthamiana* (Mogga et al. 2016; Guo et al. 2019); and the effector genes *BAS1*, *BAS2*, and *BAS4* (Bohnert et al. 2004; Mosquera et al. 2009). M5 includes *AVR-Pita1* and *BAS3* (Mosquera et al. 2009), while M6 includes *SPD2*, encoding a suppressor of cell death (Sharpee et al. 2017). Necrotrophy-associated effectors are represented in later modules, such as the necrosis-inducing protein (NLP) gene *NLP1* in M10 (Mogga et al. 2016).

We defined a total of 863 putative *Magnaporthe*effector protein (*MEP*) genes that are differentially expressed during plant infection and co-expressed with many previously reported effector genes (Supplemental Data Sets S6 and S7). To test the hypothesis that these genes encode fungal effectors, we used 2 algorithms designed to predict effector-like genes, the EffectorP algorithm and the DeepRedEff algorithm (Kristianingsih and MacLean 2021; Sperschneider and Dodds 2022). A total of 684 genes were predicted to be effector-encoding using EffectorP 3.0 and 849 genes using the DeepRedEff algorithm, based on analysis of the entire *M. oryzae* predicted proteome (Fig. 3C). In total, 546 host-induced *MEP* genes are predicted to encode effectors based on at least one of the algorithms (Fig. 3C). However, 317 *MEP* genes were not predicted to encode effectors based on either algorithm. We found that 442 predicted MEP effectors do not contain any PFAM domains. The products of *MEP4, MEP12*, and *MEP20*, however, contain a C2H2 zinc finger domain, and *MEP31* encodes a predicted secreted homeodomain protein. *MEP12* and *MEP20* were independently identified as *HTR1* and *HTR2*, which encode nuclear effectors that modulate host immunity via transcriptional reprogramming (Kim et al. 2020). *MEP24* was also reported to be involved in pathogenesis based on the finding that its overexpression led to reduced virulence (Wu et al. 2015). *MEP161* has been described as *SVP1*, encoding an *in planta*-expressed virulence effector (Shimizu et al. 2019), and *MEP11* is an allele of *AVR-Pik* (Yoshida et al. 2009). We propose that all original gene names should be given priority in the naming of these effector genes. We conclude that *M. oryzae* strain Guy11 has at least 546 effector genes and potentially as many as 863, which we have classified as differentially expressed *MEP*s.

### *MEP* genes are unevenly distributed on the chromosomes of *M. oryzae* strain Guy11

To investigate the distribution of the differentially expressed *MEP* genes in the *M. oryzae* genome, we investigated their chromosomal distribution (Supplemental Fig. S9). The full potential repertoire of 863 *MEP* genes was present at loci dispersed across all 7 chromosomes of *M. oryzae*. There appeared to be enrichment in *MEP* loci in sub-telomeric regions of all chromosomes except chromosome 5, as shown in Fig. 3D. *BAS4*, *MEP4*, *MEP7*, *MEP11*, *MEP13*, and *MEP24*, for example, are located near telomeres, and their enrichment is statistically significant (Fig. 3D). We also identified *MEP* genes that were adjacent to one another. For example, *MEP5* and *AVRPITA* are tightly linked in the sub-telomeric region of 1 arm of chromosome 6 (Supplemental Fig. S9).

### Structurally conserved *M. oryzae* effectors are temporally co-regulated during plant infection

Fungal effectors seldom show sequence similarity, or homology to known proteins, but there is increasing evidence that they may share structural conservation. Indeed, structural biology has contributed significantly to our understanding of effector evolution and function (Franceschetti et al. 2017). For example, the MAX effectors in *M. oryzae* are not related by sequence similarity but share a common β-sandwich fold that is also found in some effectors from the wheat tan spot pathogen *Pyrenophora tritici-repentis* (de Guillen et al. 2015). MAX effectors include *M. oryzae* AVR1-CO39 and AVR-PikD, which interact with heavy metal-associated (HMA) integrated domains in the immune receptors by which they are perceived during incompatible interactions (De la Concepcion et al. 2018; Guo et al. 2018).

**Figure 4.**
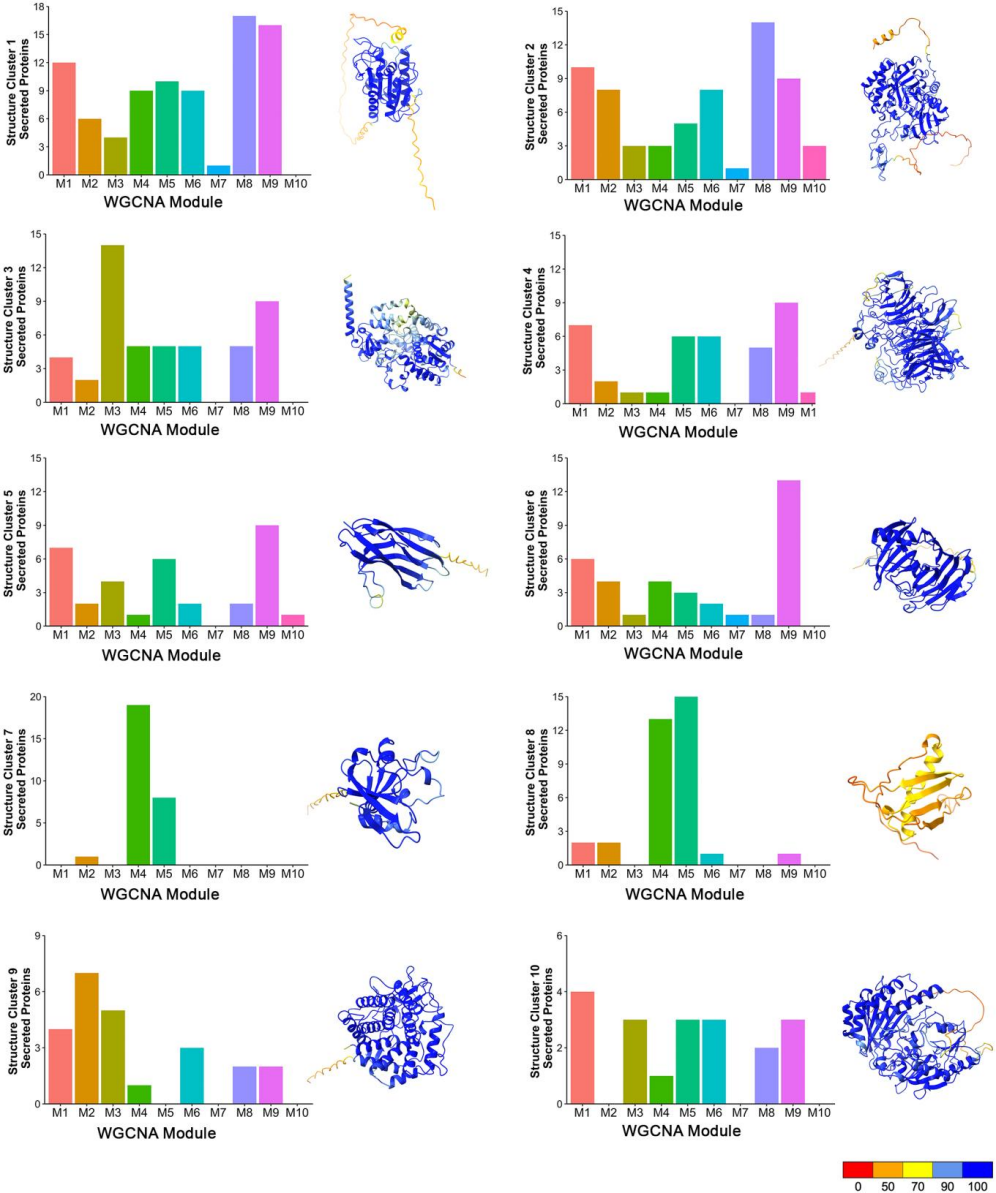
Structurally conserved *M. oryzae* effectors are temporally co-expressed during biotrophic invasive growth. AlphaFold and ChimeraX platforms were used to predict the 3D structures of predicted secreted proteins of *M. oryzae.* Structure clusters were then analyzed against each WGCNA module. Bar charts represent the number of predicted secreted proteins identified in each predicted structure cluster (Clusters 1–10), which are classified within each WGCNA co-expression module during pathogenesis. Ribbon diagrams show a representative predicted protein structure for each *M. oryzae* structure cluster. These include MGG_09840, which contains a cutinase domain for Structure Cluster 1; MGG_04534 contains a chitin recognition protein domain for Structure Cluster 2; MGG_07497 contains a cytochrome P450 domain for Structure Cluster 3; MGG_05785 (Inv1/Bas113) contains a glycosyl hydrolase family 32 domain for Structure Cluster 4; MGG_02347 (Nis1) for Structure Cluster 5; MGG_08537 contains a glycosyl hydrolase family 12 domain for Structure Cluster 6; MGG_00230 (Mep2) is a predicted ART containing a heat-labile enterotoxin alpha chain domain for Structure Cluster 7; MGG_12426 is a predicted MAX effector and homologue of AvrPib for Structure Cluster 8; MGG_04305 contains a glycosyl hydrolase family 88 domain for Structure Cluster 9; MGG_00276 contains berberine and berberine-like domains for Structure Cluster 10. Protein structures were predicted by AlphaFold, and ChimeraX was used to visualize the protein structure using PDB files generated by AlphaFold. The command “color bfactor palette alphafold” was used to color-code the confidence for each prediction (scale from red = 0% confident to blue = 100% confident). Genes encoding proteins in Structure Clusters 7 and 8 are over-represented in WGCNA M4 and M5.

Recently, a genome-wide computational structural analysis of the secreted proteome of *M. oryzae* has been reported (Seong and Krasileva 2021b), taking advantage of the development of de novo folding algorithms, such as Alphafold (Jumper et al. 2021). A total of 1,854 secreted proteins were classified into 905 structure clusters, 740 of which are represented by single proteins (Seong and Krasileva 2021b). We decided to determine the relationship between these previously reported structurally conserved families (Seong and Krasileva 2021a) and the predicted MEP effector repertoire. We found that 863 MEP proteins we grouped into 366 predicted structural clusters (Supplemental Data Set S7). These include 38 predicted MAX effectors, including Mep3, Mep11 (AVRPikC), Mep19, and Mep27, which are classified in Structural Cluster 8. We then selected the 10 largest structurally conserved clusters and analyzed the distribution of their encoding genes in each WGCNA module, as shown in Fig. 4. In most cases, the structurally conserved proteins were expressed throughout pathogenesis, but some striking patterns were observed. First of all, the MAX effector group (Cluster 8) was over-represented in M4 and M5, along with Structural Cluster 7 proteins, which are predicted ADP-ribose transferases (ARTs) proposed to act as effectors (Seong and Krasileva 2021b). This was a very distinct pattern from all of the other structurally conserved groups, which were much more evenly distributed. The other pattern observed was some over-representation of predicted hydrolases (Cluster 1), glucosidases (Cluster 2), and glycosyl hydrolases (Clusters 4 and 6) in M8 and M9, which are associated with necrotrophy. We conclude that structurally conserved effector candidates are temporally co-expressed during plant infection.

### Stage-specific expression of Mep effectors of *M. oryzae*

**Figure 5.**
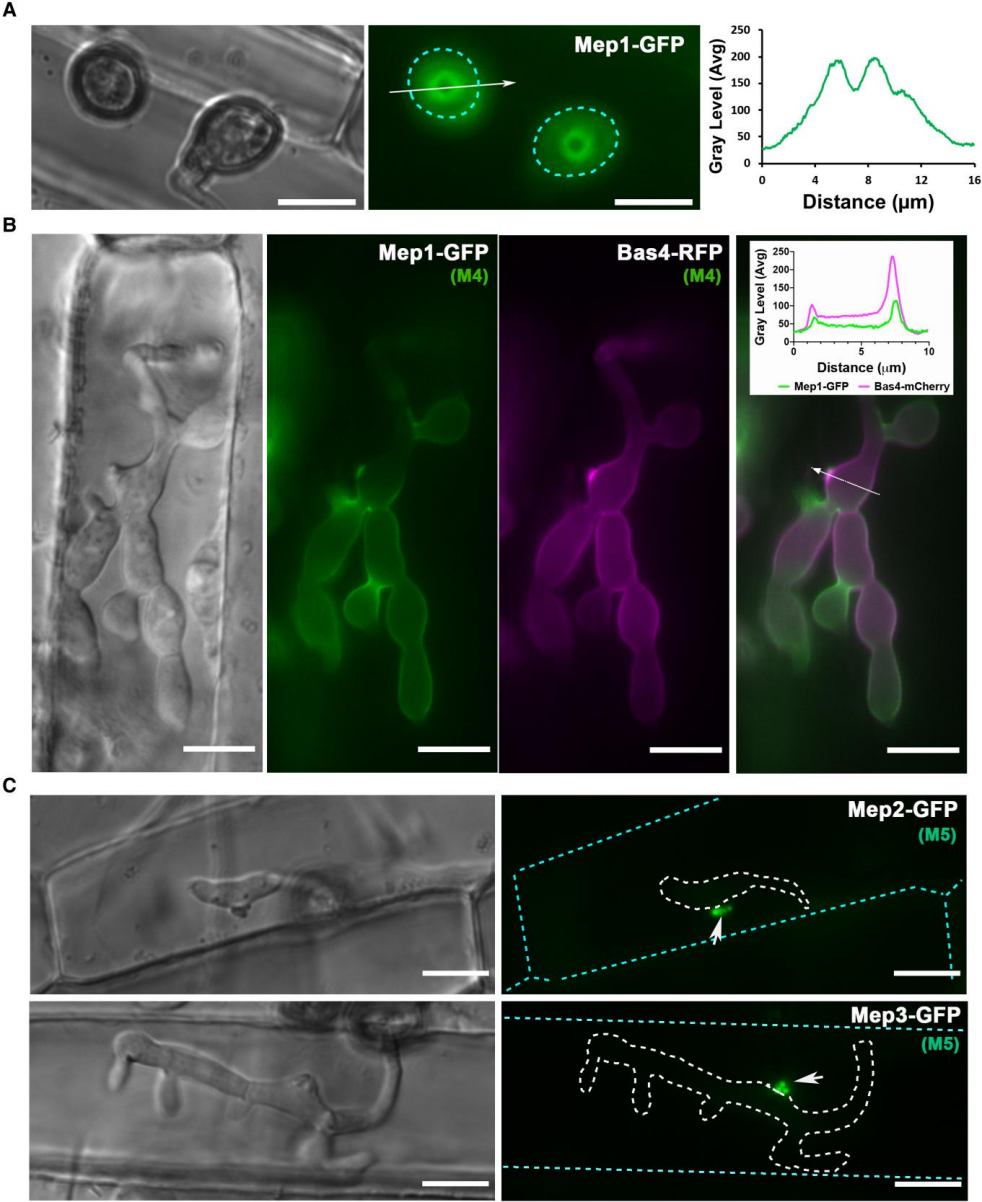
Live-cell imaging of Mep candidates during plant infection reveals spatial localization of effectors during appressorium penetration and invasive growth. **A)** Micrographs showing the M4 effector Mep1-GFP localizing in a ring conformation at the appressorium pore at the leaf surface in *M. oryzae* Guy11. Conidial suspensions at 5 ×10^4^ mL^−1^ were inoculated onto rice leaf sheath and images captured at 24 hpi. The periphery of the appressorium is indicated by a cyan dotted line. Line scans show Mep1-GFP fluorescence in a transverse section of the appressorium. Scale bars = 10 µm. **B)** Conidia were harvested from a *M. oryzae* transformant expressing Mep1-GFP and Bas4-RFP gene fusions and inoculated onto rice leaf sheath preparations. Images were captured at 28 hpi of invasive growth. Micrograph and line scan graphs show co-localization of Mep1-GFP and Bas4-RFP in invasive hyphae of the *M. oryzae* wild-type strain Guy11. Arrow indicates the area used for line scan analysis. Scale bars = 10 µm. **C)** Micrographs showing the localization of representative M5 effector candidates Mep2-GFP and Mep3-GFP from the 32 selected Mep proteins (Supplemental Fig. S10 and Supplemental Data Set S8) during plant infection. Images were captured at 22 to 24 hpi. Invasive hyphae are outlined by a white dotted line and individual plant cells indicated with a cyan dotted line. Short white arrows indicate localization of both Mep2-GFP and Mep3-GFP at the BIC. Scale bars = 10 µm.

In order to focus on the most likely effector candidates for experimental analysis, we randomly selected 32 *MEP* genes identified in the CO39 leaf drop experiment (Fig. 3C and Supplemental Data Sets S3–S5) and characterized them by live-cell imaging and targeted gene replacement. We first generated *GFP* (green fluorescent protein gene) fusions of the 32 representative *MEP* genes, expressed these GFP fusions in Guy11, and visualized their sub-cellular localization patterns during fungal growth *in planta*. A sub-set of effectors could be visualized very early during plant infection. *MEP1*, for example, is organized into a ring conformation at the appressorium pore on the plant cell surface (Fig. 5). The appressorium pore is organized by septin GTPases and marks the point of penetration peg emergence and polarized exocytosis, a process mediated by the exocyst complex (Gupta et al. 2015). Mep1 is, therefore, likely to be secreted at the point of cuticle penetration from the penetration peg. After cuticle rupture, Mep1-GFP initially accumulates at the tip of the primary invasive hypha and then outlines invasive hyphae, suggesting that it localizes to the apoplast between the fungal cell wall and the plant plasma membrane, termed the Extra-Invasive Hyphal Membrane (EIHM) (Kankanala et al. 2007), as shown in Fig. 5B and Supplemental Movie 7. Consistent with this idea, Mep1-GFP co-localizes with Bas4-RFP, an apoplastic effector (Giraldo et al. 2013) around invasive hyphae 24 h after infection, which is consistent with its classification in M4 (Fig. 5B). By contrast, the M5 candidate effector Mep3 localizes exclusively to the BIC during infection, with GFP signal peaking at 48 h (Fig. 5C). Exclusive BIC localization was observed for all other Mep-GFP fusions examined within the initial cell penetrated (Supplemental Fig. S10).

**Figure 6.**
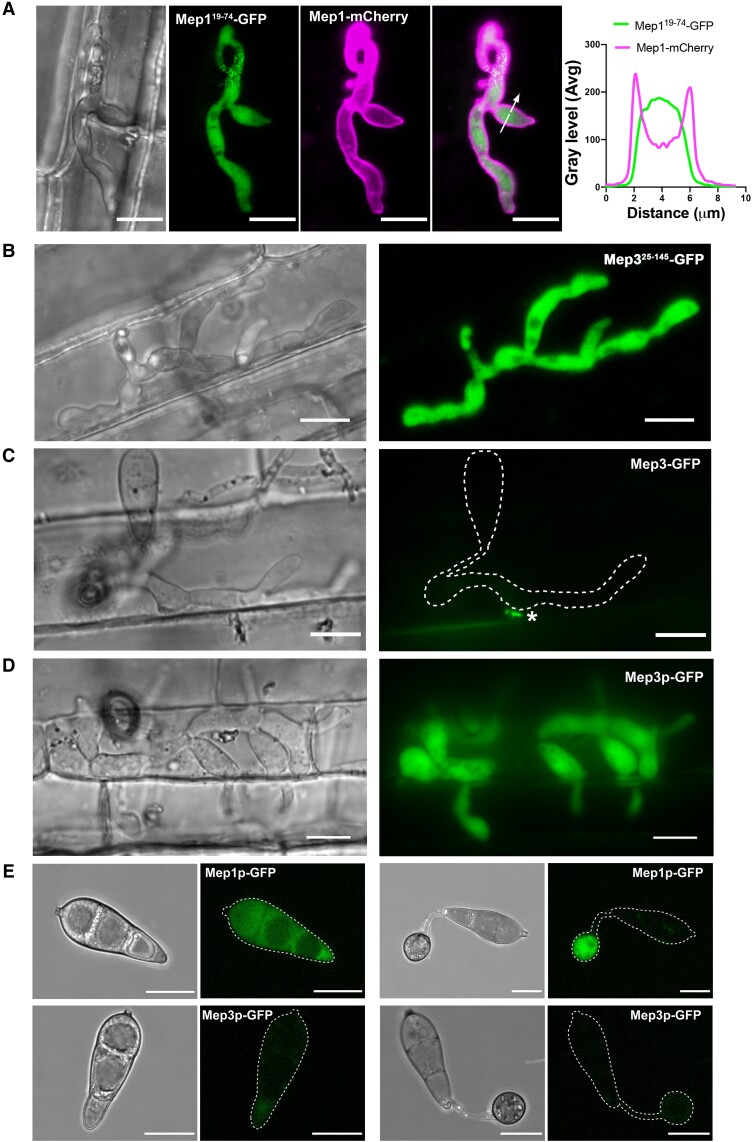
*MEP* genes are highly expressed in living plant tissue during plant infection. **A)** Conidia were harvested from a *M. oryzae* transformant expressing a Mep1^Δ19–74^-GFP signal peptide deletion and a Mep1-RFP full-length gene fusion and inoculated onto CO39 rice leaf sheath. Images were captured at 24 hpi of invasive growth. GFP fluorescence (in green) was retained inside the invasive hyphae, while mCherry fluorescence (in magenta) was exported to the apoplast. Micrograph and line scan graphs show that the Mep1 signal peptide is required for correct protein secretion. Arrow indicates the area used for line scan analysis. Scale bars = 10 µm. **B)** Micrographs showing a rice plant infected by the *M. oryzae* wild-type strain Guy11 expressing a Mep3^Δ25–145^*-*GFP signal peptide deletion. Images were captured at 24 hpi of invasive growth. Scale bars = 10 µm. **C)** Micrographs showing rice plant tissue infected by the *M. oryzae* wild-type strain Guy11 expressing full-length *MEP3,* driven by its native promoter. Mep3-GFP could be observed as small puncta at the BIC. Asterisk indicates the BIC. Images were captured at 24 hpi of invasive growth. **D)** Micrographs showing rice CO39 infected with *M. oryzae* wild-type strain Guy11 expressing cytoplasmic GFP driven by the *MEP3* promoter. Scale bars = 10 µm. **E)** Conidia were harvested from *M. oryzae* transformants expressing GFP driven by the promotor of Mep1p-GFP and Mep3p-GFP, respectively, and inoculated onto hydrophobic glass coverslips. Micrographs of Mep1p-GFP showed fluorescent signal in both the conidium and the appressorium, in contrast to Mep3p-GFP, which displayed little signal. Appressorium formation was observed at 20 hpi. Scale bars = 10 µm.

To investigate the secretion of MEP effectors, we generated alleles in which the predicted signal peptide region was removed and expressed these in *M. oryzae*. When Mep1^19–74^-GFP was expressed with Mep1-mCherry in Guy11, we observed cytoplasmic accumulation of Mep1^19–74^-GFP, while Mep1-mCherry was delivered to the apoplast around invasive hyphae (Fig. 6A and Supplemental Fig. S11). The signal peptide of cytoplasmic effectors was also necessary to enable delivery to the BIC, with Mep3^25–145^-GFP fluorescence observed intracellularly in invasive hyphae (Fig. 6B). Because the punctate localization of BIC effectors might be interpreted as being a lower expression level than predicted by RNA-seq analysis (Fig. 6C), we also constructed gene fusions expressing free GFP driven by each *MEP* promoter, as shown for *MEP1* and *MEP3* in Fig. 6, D and E. Fluorescence was observed for *MEP1* in conidia and appressoria, as well as invasive hyphae, while *MEP3* was expressed at a very high level exclusively in invasive hyphae (Fig. 6, D and E and Supplemental Fig. S12). Cell biological visualization of MEP expression was, therefore, consistent with predictions from RNA-seq analysis.

### The dynamics of the host–pathogen interface during plant infection

**Figure 7.**
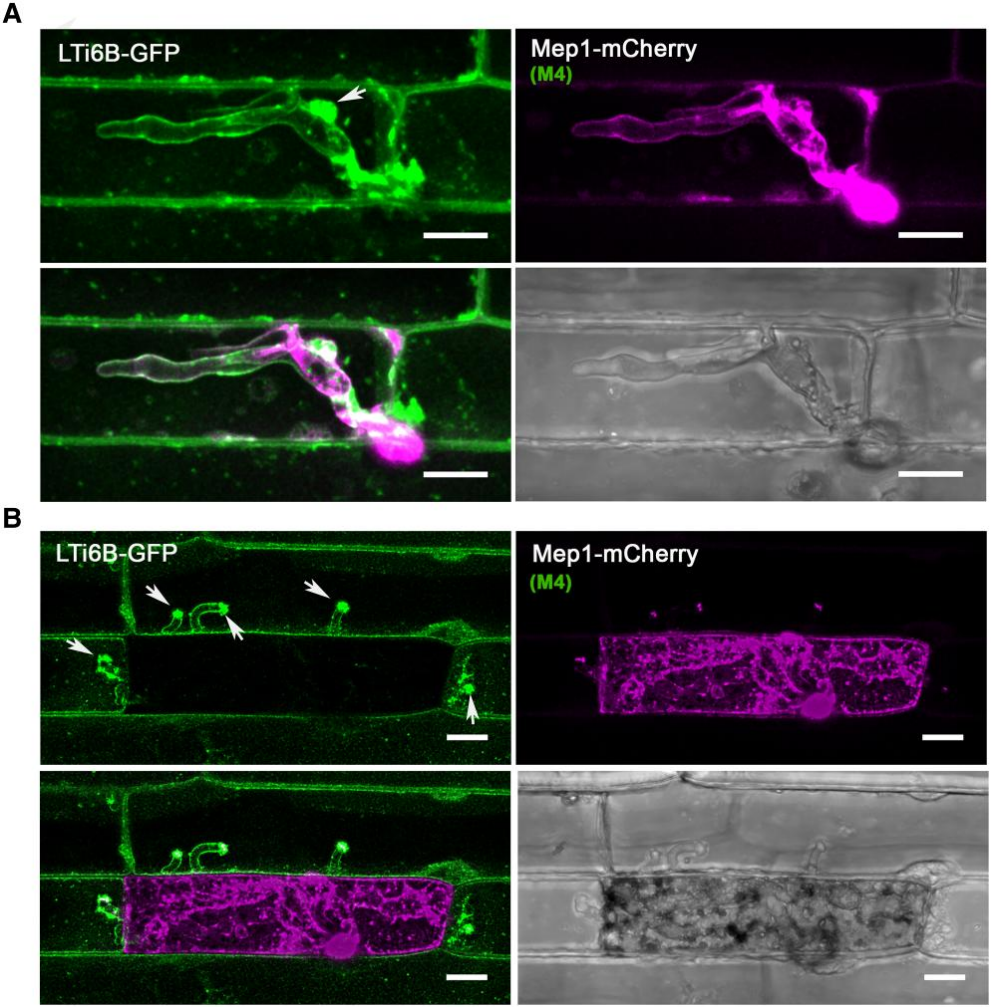
The rice plasma membrane is invaginated and accumulates at the BIC during plant infection. Laser confocal micrographs of *M. oryzae* expressing Mep1-mCherry colonizing epidermal leaf cells of a transgenic rice line expressing plasma membrane-localized LTi6B-GFP. Images were captured at 24 hpi **(A)** and 36 hpi **(B)**. The plant plasma membrane stays intact and invaginated at the early stages of plant infection. LTi6B fluorescence accumulates at the bright BIC, indicating the BIC is a plant membrane-rich structure. The fluorescence signal from secreted Mep1-mCherry is surrounded by the fluorescence signal of the rice cell plant plasma membrane marker LTi6B-GFP as the fungus invades new cells, but the initial epidermal cell is occupied and then loses viability, and Mep1-mCherry fluorescence fills the rice cell. Arrows indicate the BICs in the invaded neighboring cells. Scale bars = 10 um.

To investigate the dynamics of the host–pathogen interface during plant infection, we infected a transgenic rice line expressing the plasma membrane marker Lti6b-GFP (Kurup et al. 2005; Mentlak et al. 2012) with a Guy11 strain expressing Mep1-mCherry. The Mep1-mCherry fluorescence outlined invasive hyphae in an initially colonized epidermal cell, bounded by the EIHM, which showed Lti6b-GFP fluorescence, consistent with delivery of the effector to the apoplast between the fungal cell wall and EIHM. The EIHM maintained integrity during the early stages of plant infection (Fig. 7A), but membrane integrity was lost once the fungus invaded neighboring cells, resulting in the loss of host cell viability (Fig. 7B). The Mep1-mCherry signal accumulated in the first invaded cell, suggesting rupture of the plant plasma membrane and closure of plasmodesmata. Meanwhile, new BICs formed immediately upon entry into neighboring cells enriched with plant membrane.

### Mep effectors secreted to BICs are always delivered into host cells

**Figure 8.**
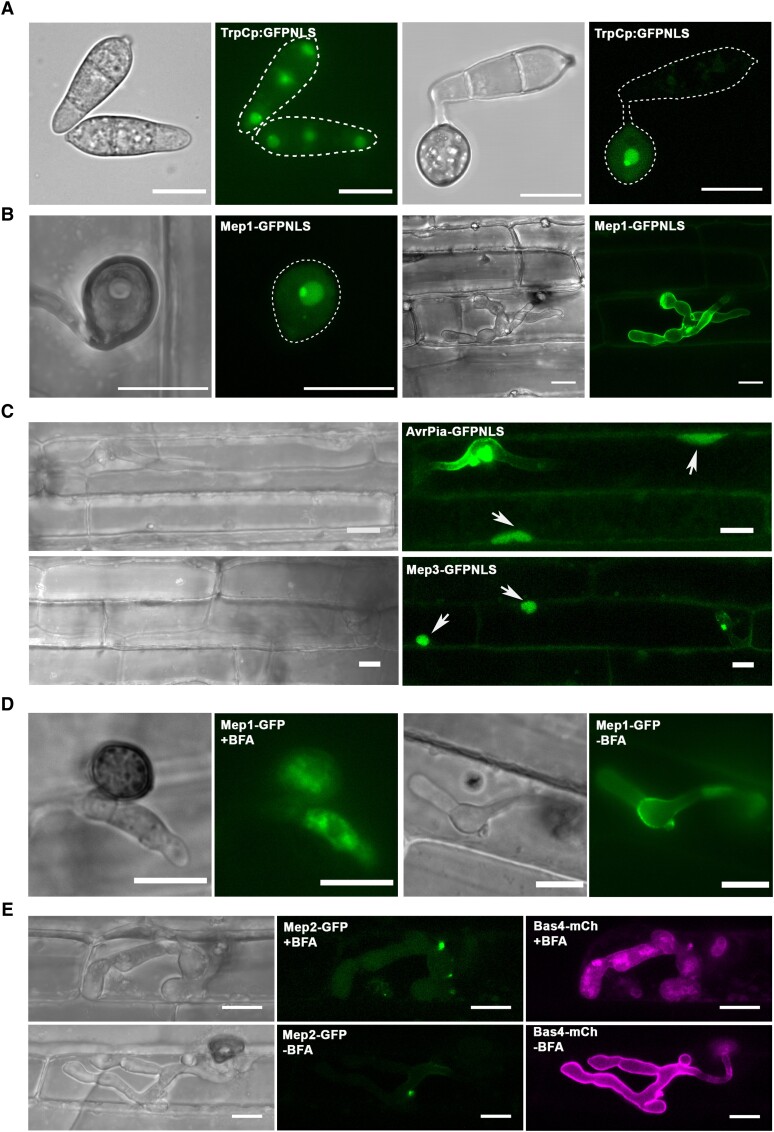
Cytoplasmic Mep candidates, which accumulate at the BIC, are translocated into host cells. **A)** Cellular localization of GFP with an NLS driven by the TrpC promoter in *M. oryzae*. The fluorescence signal in the nuclei and nucleoli of conidia and the appressorium. The outline of the fungus is depicted by a white dotted line. **B)** Micrographs of Mep1-GFP^NLS^ inoculated onto rice leaf sheath and captured at 28 hpi. Fluorescence could be observed in the nucleus and nucleolus of the appressorium, but not in the fungus or plant cells. **C)** Micrographs of AvrPia-GFP^NLS^ and BIC-accumulating Mep effector candidates showing delivery into both the invaded host cell and unoccupied neighboring host cells. Arrows indicate plant nuclei. **D)** Cellular localization of Mep1-GFP in *M. oryzae* during biotrophic growth on epidermal rice cells. BFA was applied at 20 hpi, and 0.1% DMSO was used for the control treatment. Images were captured at 3 to 4-h posttreatment. Mep1-GFP fluorescence shows accumulation within invasive hyphae and particularly in the BIC-associated cell. **E)** Micrographs of *M. oryzae* expressing Mep2-GFP and Bas4-mCherry during biotrophic growth on epidermal rice cells. BFA treatment was used to examine the secretion of Mep2-GFP and Bas4-mCherry. Mep2-GFP fluorescence accumulated in the BIC in the presence or absence of BFA. By contrast, Bas4-mCherry fluorescence accumulated inside invasive hyphae. Scale bars = 10 µm.

Translocation of effectors into rice cells has previously been visualized by expressing fluorescent effector fusion proteins in *M. oryzae* with an artificially added C-terminal nuclear localization signal (NLS) (Khang et al. 2010). The NLS allows effectors to be detected in plant cells by concentrating them in the rice nucleus. This assay has previously been used, for example, to show that the PWL2 effector, which acts as a host specificity determinant in *M. oryzae,* is translocated into rice cells (Khang et al. 2010). To determine whether *MEP*-encoded effectors are translocated into host cells, we generated translational fusions with the *Mep* effector gene fused in-frame to an NLS. As a control, we expressed GFP-NLS driven by the constitutive TrpC promoter and found that the GFP signal accumulated predominantly in fungal nuclei, confirming that GFP-NLS is functional and can drive free GFP into nuclei. However, unless specifically delivered, the signal will not move into a plant nucleus during plant infection (Fig. 8A).

To investigate whether the Mep1 effector, which we localized to the apoplast, can be translocated into host cells, we expressed Mep1-GFP-NLS in *M. oryzae* and infected rice seedlings with this fungal transformant. Even though the effector possessed a signal peptide and normally localizes to the appressorium pore (as shown in Fig. 5A), fluorescence was observed predominantly in the fungal nucleus (Fig. 8B). No GFP signal was observed in the plant nucleus of the occupied rice cell or in surrounding plant nuclei (Fig. 8B). By contrast, when the BIC-localized effector Mep3 was fused to an NLS, the Mep3-GFP-NLS signal was observed in the plant nucleus of the occupied cell and those of adjacent cells (Fig. 8C). All BIC-localized effectors evaluated using this assay showed translocation to host cells, while apoplastic effectors, such as Mep1, were not translocated.

Apoplastic and cytoplasmic effectors of *M. oryzae* have been reported to be secreted by different pathways during rice infection, but to date, only a very limited sample has been analyzed (Giraldo et al. 2013). Cytoplasmic effectors such as Pwl2 were shown to be secreted in a brefeldin A (BFA)-insensitive manner, suggesting that they do not undergo conventional endoplasmic reticulum-to-Golgi secretion. By contrast, apoplastic effectors such as Bas4 are secreted conventionally and are sensitive to BFA treatment. We, therefore, applied BFA to rice tissue infected with *M. oryzae* expressing Mep-GFP fusions. The secretion of Mep1-GFP was significantly inhibited in the presence of BFA, with fluorescence accumulating within invasive hyphae (Fig. 8D). However, when we assayed BIC-associated effectors such as Mep2 and Mep3, we found they were BFA-insensitive. When a *M. oryzae* Guy11 isolate expressing Mep2-GFP and Bas4-mCherry was exposed to BFA (Fig. 8E), the BAS4-mCherry signal was prevented from being delivered to the apoplast, while the Mep2-GFP signal remained in the BIC. This was observed for all putative effectors that were analyzed (Supplemental Data Set S8). We conclude that Mep effectors are secreted by 2 distinct secretory pathways, depending on their host destination (Giraldo et al. 2013).

### Mep effectors contribute to pathogen fitness

Pathogenic fungi secrete a large battery of effector proteins during infection, but individual effector genes have often been reported to display no discernable mutant phenotype with regard to virulence. This has been interpreted as being a consequence of functional redundancy and overlapping effector functions, such that the role of an individual effector is very hard to measure (Selin et al. 2016). In *M. oryzae*, most effectors characterized to date—such as Pwl2, Bas1, Bas2, Avr-Pik, and Avr-Pita—do not contribute substantially to the ability of the fungus to cause disease. A much smaller number of effectors, such as the extracellular LysM effector Slp1, do contribute to fungal virulence (Mentlak et al. 2012), but such reports are very rare. However, an increasing number of studies indicate that effectors serve important functions in the suppression of plant immunity. Effectors interfere with the operation of pattern recognition receptors, impair host responses such as reactive oxygen species generation, and suppress immunity signaling pathways and transcriptional responses (Irieda et al. 2019; Kim et al. 2020).

**Figure 9.**
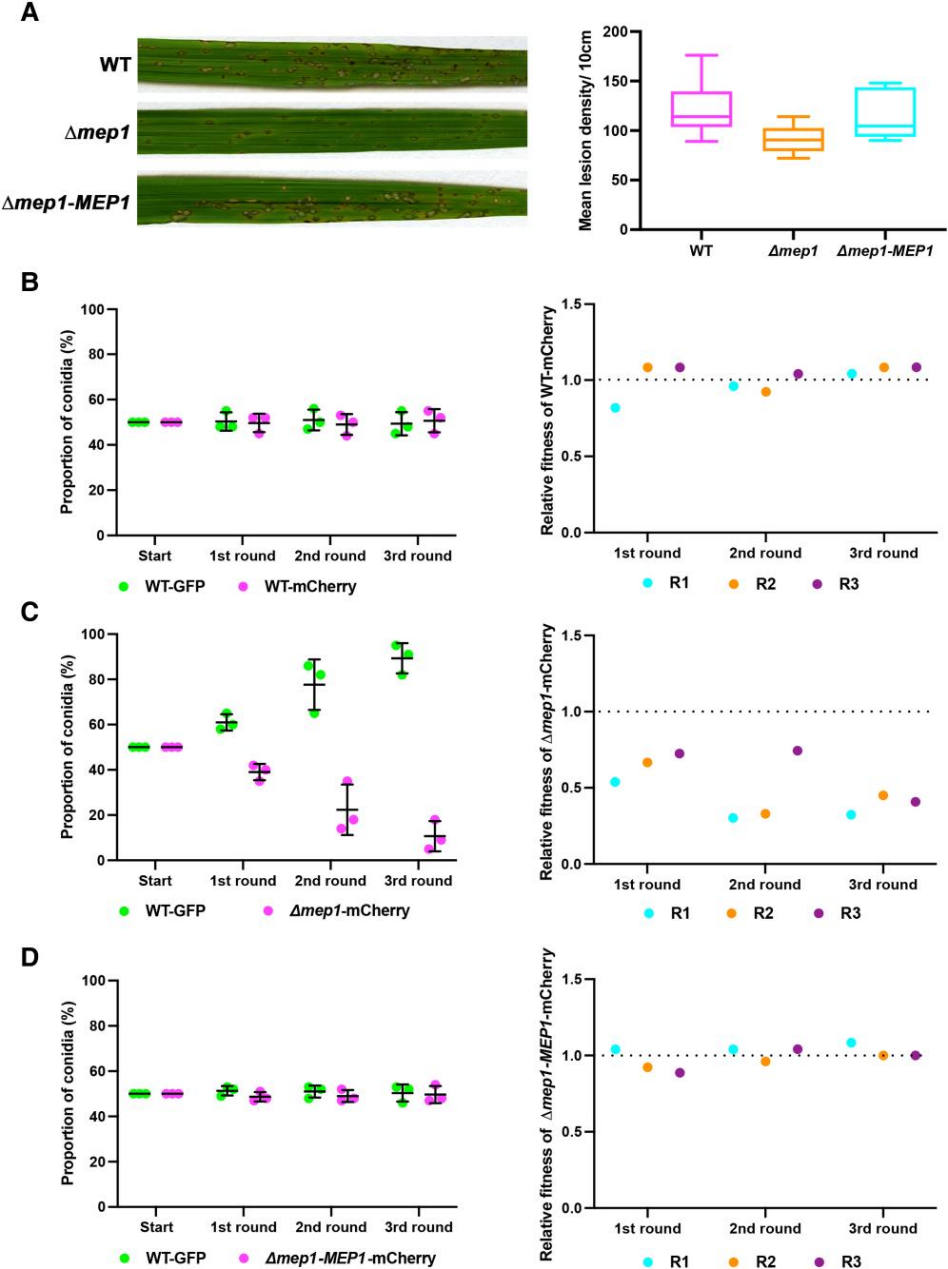
Mep1 contributes to pathogen fitness during plant infection. **A)** Conidial suspensions of equal concentration (5 × 10^4^ spores mL^−1^) from *M. oryzae* Guy11, Δ*mep1*, or Δ*mep1* complementation strain Δ*mep1-MEP1* were used to inoculate 21-d-old seedlings of the blast-susceptible cultivar CO39 and disease symptoms recorded after 5 dpi. The box plot shows the lesion density of seedlings infected with Guy11 and the Δ*mep1* mutant per unit area. The lower horizontal line shows the minimum value, and the upper horizontal line shows the maximum value. The lower border and upper border of the box show the lower quartile and upper quartile, respectively. The line in the box shows the median. Whiskers showing Min to Max. **B–D)** A relative fitness assay was carried out by mixing conidia in equal amounts (1:1) from Guy11 expressing GFP (WT-GFP) vs. Guy11 expressing mCherry (WT-mCherry), Guy11 expressing GFP (WT-GFP) vs. *Δmep1* mutant expressing mCherry (*Δmep1*-mCherry), and Guy11 expressing GFP (WT-GFP) vs. the complemented strain of *Δmep1* expressing mCherry (*Δmep1*-*MEP1*-mCherry). Spores were collected from disease lesions and used to inoculate new seedlings in the recovered proportions. **B)** The dot plot on the left shows the proportion of Guy11 TrpCp:GFP (WT-GFP) and the Guy11 TrpCp:mCherry (WT-mCherry) conidia recovered from each generation. Bars represent the mean ± SD of 3 replicates. The dot plot on the right shows the relative fitness of Guy11 TrpCp:mCherry. **C)** The dot plot on the left shows the proportion of Guy11 TrpCp:GFP (WT-GFP) and Δ*mep1* TrpCp:mCherry (*Δmep1*-mCherry) conidia recovered from each generation. The Δ*mep1* TrpCp:mCherry mutant was driven to near extinction in 3 generations. Bars represent the mean ± SD. The dot plot on the right shows the relative fitness of strain Δ*mep1* TrpCp:mCherry. **D)** The dot plot on the left shows the proportion of Guy11 TrpCp:GFP (WT-GFP) and the Δ*mep1*-*MEP1* TrpCp:mCherry (Δ*mep1*-*MEP1*-mCherry) conidia recovered from each generation. Bars represent the mean ± SD. The dot plot on the right shows the relative fitness of the complemented Δ*mep1*-*MEP1* TrpCp:mCherry strain. Relative fitness was calculated using the equation *x*2(1 − *x*1)/*x*1 (1 − *x*2), where *x*1 is the initial frequency of conidia from the tested strain and *x*2 is the final frequency.

The contribution of an effector to fungal virulence is normally evaluated by generating a targeted deletion mutant and infecting a susceptible host cultivar. Disease symptoms are then measured and compared with an isogenic wild-type strain. We observed that infections with Δmep1 mutants led to reduced disease symptoms (Fig. 9A and Supplemental Fig. S13). We decided, however, that this assay was likely to be insufficiently sensitive to be able to accurately determine the actual contribution of a given Mep effector to rice blast disease. We, therefore, deployed a relative fitness assay (Ross-Gillespie et al. 2007; Lindsay et al. 2016) to evaluate the contribution of an effector to fungal virulence. We generated a Guy11 strain expressing cytoplasmic GFP under control of a high-level constitutive promoter, TrpC. At the same time, we generated a second Guy11 strain expressing cytoplasmic mCherry driven by the same TrpC promoter, as well as the Δmep1 mutant (Supplemental Fig. S14), also expressing cytoplasmic mCherry driven by the same TrpC promoter. In a control experiment, we generated a spore inoculum in which we mixed Guy11 TrpCp:GFP conidia with Guy11 TrpCp:mCherry conidia in a 1:1 ratio. We then prepared an inoculum containing a 1:1 ratio of Guy11 TrpCp:GFP conidia and Δmep1 TrpCp:mCherry conidia, and finally, an inoculum containing a 1:1 ratio of Guy11 TrpCp:GFP conidia with conidia from the complemented Δmep1-*MEP1* TrpCp:mCherry strain of *M. oryzae.* In all cases, the inocula were used to infect CO39 seedlings. We allowed the disease to progress and then recovered conidia from disease lesions. We recorded the ratio of spores recovered and then used the same ratio to prepare inoculum to infect a second batch of rice seedlings. We repeated the assay for each generation.

In the control experiment using Guy11 strains, we observed no significant difference in the ratio of GFP and mCherry conidia recovered after 3 generations (Fig. 9B). By contrast, in a mixed infection with Guy11 TrpCp:GFP conidia and Δmep1 TrpCp:mCherry conidia, the Δmep1 mutant strain was driven close to extinction after 3 rounds of infection (Fig. 9C). Finally, reintroduction of the *MEP1* gene into a Δmep1-*MEP1* TrpCp:mCherry strain complemented the observed fitness defect (Fig. 9D). We calculated relative fitness using the equation *x*2(1 − *x*1)/*x*1 (1 − *x*2), where *x*1 is the initial frequency of the Mep mutant in the population and *x*2 is the final frequency after 3 generations of infection (Fig. 9, B–D). In a further control experiment, we mixed wild type and Δmep1 mutant conidia in axenic plate culture and then recovered spores 10 d later. We observed that they maintained the same 1:1 ratio, showing that Δmep1 mutants have equivalent fitness when growing outside of a plant (Supplemental Fig. S14). When considered together, these results suggest that Mep1 contributes to the ability of *M. oryzae* to cause rice blast disease, and its loss has a significant effect on the relative fitness of the fungus at a population level. In this way, we were, therefore, able to calculate the cost of losing an individual effector during plant infection.

## Discussion

The ability of the rice blast fungus to colonize plant tissue and cause disease is still relatively poorly understood (Fernandez and Orth 2018; Eseola et al. 2021). The most significant recent advances have come from exploring the cellular changes that accompany fungal infection (Khang et al. 2010; Eseola et al. 2021), the regulation of primary metabolism associated with biotrophic growth (Sun et al. 2018), the secondary metabolic pathways associated with the suppression of host immunity (Patkar et al. 2015; Marroquin-Guzman et al. 2017), the definition of effector functions (Mentlak et al. 2012; Kim et al. 2020), and the identification of signaling pathways associated with invasive growth (Sakulkoo et al. 2018). These studies have provided insight into the substantial changes elicited by *M. oryzae* as it infects rice plants in order to cause disease (Fernandez et al. 2014; Cruz-Mireles et al. 2021). These advances have been coupled with numerous studies to validate the roles of individual genes in pathogenesis, although these have predominantly been associated with appressorium-mediated infection, with only a small number of genes defined as being determinants of tissue colonization (Fernandez and Orth 2018). Our understanding of the biology of rice tissue colonization has, therefore, been limited due to a lack of holistic studies of blast infection. The motivation for this study was to carry out transcriptional profiling of the entire disease cycle of *M. oryzae* in order to reveal fundamental changes in pathogen gene expression that occur during the progression of the disease and to use this information to identify the full repertoire of fungal effector proteins deployed by the fungus.

Transcriptional profiling has been used to investigate several plant–pathogen interactions to provide insight into fungal development inside a host plant (Kawahara et al. 2012; Kleemann et al. 2012; Rudd et al. 2015; Dobon et al. 2016; Wang et al. 2017; Lanver et al. 2018). These studies have suggested that the expression of effector proteins is an emerging characteristic of fungal invasion of plant hosts. In the corn smut fungus *Ustilago maydis*, transcriptional profile analysis has provided significant insight into the biology of biotrophic development. Distinct temporal expression profiles have, for instance, been defined for a wide range of genes associated with fungal metabolism, nutrient acquisition, regulatory networks, and secrete effectors. In particular, *U. maydis* effectors were found to be expressed in 3 distinct expression modules associated with growth on the leaf surface, biotrophic development in maize cells, and during the induction of tumor formation, when rapid plant cell division is stimulated (Lanver et al. 2018). These findings highlight the need for studies at such a level of resolution in *M. oryzae*.

The most comprehensive study to date in *M. oryzae* used laser capture microdissection to enhance the proportion of rice cells containing invasive fungal hyphae and then employed microarray analysis to define a total of 58 putative fungal effectors, 4 of which were localized during the infection process (Mosquera et al. 2009). A more recent *M. oryzae* study using RNA-seq analysis focused on a single time point of infection, 24 hpi, when rice cells were initially occupied by the fungus. This led to the identification of 240 fungal transcripts, which included some effector candidates (Kawahara et al. 2012). These studies of *M. oryzae*, although influential, had severe limitations in their coverage of the development of rice blast and the level of resolution they were able to achieve. Laser capture microdissection, for example, added a layer of perturbation to sample preparation (Mosquera et al. 2009), while focusing on a single time point (Kawahara et al. 2012) limited the identification of effectors. We, therefore, decided to perform a comprehensive transcriptional profiling study in which 8 time points would be used, along with distinct inoculation methods and different fungal–rice cultivar interactions, to identify the maximum number of fungal transcripts and provide the closest relationship to natural blast infections.

The major findings of our study are (i) the definition of 10 distinct modules of differentially regulated *M. oryzae* genes that encompass the entire process of pathogenic development from the time of spore germination to the development of sporulating disease lesions; (ii) the identification of distinct physiological processes and genes involved in primary and secondary metabolism expressed at specific stages of pathogen development; (iii) the identification of *MEP* effector genes that are temporally expressed throughout pathogenesis in distinct temporal groups; (iv) the discovery that structurally conserved effectors are temporally co-regulated during invasive growth, providing a means of identifying novel effector functions; (v) the classification of Mep effectors into cytoplasmic and apoplastic proteins, delivered via 2 distinct secretory routes, validating this classification system for the effector repertoire, and (vi) the definition of Mep effector function based on their contribution to pathogen fitness using a mixed inoculation assay and fitness measure.

Temporal analysis of gene expression revealed the nature of changes in physiological function during pathogenic development. This highlighted the rapid growth of *M. oryzae* during spore germination and starvation stress associated with early development on the leaf surface. Consistent with this notion, genes associated with autophagy, regulated proteolysis, and lipid metabolism were expressed. During early biotrophic colonization of leaf tissue, genes associated with carbon and nitrogen source acquisition from the plant host were significantly upregulated by 24 h. The orchestration of secondary metabolic pathways is also a feature of the biotrophic colonization of initially occupied epidermal cells, in addition to effector secretion. Another key feature recognized during infection is the pattern of repression of gene expression that accompanies the developmental program following appressorium development. This is consistent with the notion that embryogenic specialization in cell fate constrains patterns of gene expression and may be a largely unexplored feature of pathogenesis, as so many studies have instead focused exclusively on genes induced during infection.

A pattern, therefore, emerges of how *M. oryzae* switches from a nutrient-free environment of the leaf surface where its metabolism is dominated by lipid metabolism and re-cycling of the spore contents, which is consistent with the rapid generation of compatible solutes, such as glycerol, for appressorium turgor generation. As the fungus encounters sugars and amino acids within the leaf, it switches to rapidly acquiring nutrients as it moves through rice cells. A transition to sucrose utilization and the pentose phosphate pathway is apparent, as demonstrated in experimental studies in which the *M. oryzae* glucose-6-phosphate sensor Tps1 was shown to regulate carbon metabolism and to link glucose availability to glutathione-dependent antioxidation and the establishment of biotrophy (Wilson et al. 2010; Fernandez et al. 2012). The biotrophy-associated gene *IMP1*, for example, which is linked to TOR-dependent nutritional control of invasive growth (Sun et al. 2018), is in M6 and peaks in expression at 48 h after infection. The pattern of primary metabolism-associated gene expression is also consistent with the results of a previous metabolomic analysis of plant infection by *M. oryzae* (Parker et al. 2009), which revealed fungal reprogramming of plant metabolism during infection, including suppression of the defense-associated reactive oxygen species burst. For example, the nitronate mono-oxygenase gene *NMO2*, which is required to limit nitro-oxidative stress during rice immune responses (Marroquin-Guzman et al. 2017), peaks in expression at 16 and 48 h, during the most active phases of biotrophic proliferation of the fungus. Our study also provides evidence of a very large-scale change in gene expression during the later stages of infection (after 48 h) associated with the utilization of completely distinct families of transcriptional regulators as the fungus transitions to necrotrophy and disease symptom development.

Arguably the most significant finding of this study is that the effector repertoire of *M. oryzae* is likely to be much more substantial than previously thought, with at least 546 putative effectors recognized, among a total of 863 differentially regulated secreted proteins. Effector functions are continually being described across phytopathogenic fungi, and in *M. oryzae*, a range of effector targets have been identified, including small HMA domain proteins, inhibitors of chitin-triggered immunity, and exocyst components (Franceschetti et al. 2017). In all cases described so far, the effector suppresses pattern-triggered immunity (PTI). However, the sample sizes evaluated in these studies were extremely small compared with the likely total repertoire revealed in the current study (less than 2%), suggesting that the fungus has the capacity to overwhelm plant defense, perhaps via intervention at key points, with the same targets identified by many effectors. Conversely, our findings suggest that there are many new effector functions to be discovered, including potentially some not associated with PTI suppression, perhaps inducing morphological changes in rice cells essential for pathogen development.

A limitation in fungal effector identification has been the lack of sequence similarity among effectors and the absence of specific motifs, such as the RXLR motif found in many oomycete effectors (Morgan and Kamoun 2007). The use of genome-wide computational structural biology approaches provides a powerful means of identifying putative effectors, given the structural similarity exhibited by the MAX effectors and ARTs (Seong and Krasileva 2021b). We observed that both of these structurally conserved effector families are temporally co-expressed within M4 and M5, making them the most enriched for computationally predicted fungal effectors. This has provided a wealth of effector candidates for functional analysis and also suggests that examining genes co-expressed in these 2 WGCNA modules may provide a means of mining for novel effector functions, utilizing the combination of structural prediction and temporal expression profiling. It may also be possible to combine these analyses with an evaluation of the chromosomal distribution of gene loci as an additional diagnostic tool for predicting likely effector candidates.

The study of effectors has also been constrained by the difficulty in assessing their contributions to the biology of the pathogen. This is largely a consequence of the relatively crude assays used to define their role in pathogenesis. Carrying out targeted deletion and simply assaying their ability to generate disease symptoms has not generally led to discernible mutant phenotypes. We, therefore, carried out a mixed infection assay designed to allow the relative contribution of an effector to pathogen fitness to be measured. We reasoned that if an effector makes a small contribution to the fitness of *M. oryzae* during disease development, a mutant lacking that effector will be less likely to survive within the pathogen population. We, therefore, marked strains with different fluorescent markers and produced inocula with equal numbers of spores to infect plants. This provided a simple means of assessing fitness over several generations. The assay does not provide information on why the lack of an effector affects virulence directly. This would require quantitative analysis of the rate of plant cell colonization and tissue invasion, which is technically challenging to carry out on large numbers of individual infections. Instead, the number of mutant conidia produced from infected rice plants is used as a proxy for the number of disease lesions generated by an effector mutant and their size compared to those generated by the wild type. The assay clearly has some limitations, and caution is necessary in interpretation of the results—it would not distinguish between mutants affected in penetration or conidia production from disease lesions, for instance. The simplicity of the assay, however, lies in the fact that it can be carried out at the scale of cell biological analysis, and the results enable a fitness coefficient to be calculated for an individual mutant within the pathogen population (Ross-Gillespie et al. 2007; Lindsay et al. 2016). In the case of the *MEP1* effector gene, targeted deletion resulted in a small but significant change in disease lesion generation, but in mixed infections, the Δmep1 mutant was driven to extinction in 3 generations. The assay may, therefore, have utility for evaluating effector function in the future and could be further refined by bar-coding to enable mixed populations of effector mutants to be analyzed in greater detail.

In summary, this study has provided a resource for researchers to ask specific questions regarding the major transcriptional changes associated with rice blast disease, including both repression and activation of gene functions. The analyses reported here are a very small element of what could be addressed with the data sets, which offer a deep, extensive source of transcriptome data for the rice–*M. oryzae* interaction. Furthermore, we have identified a very large battery of more than 546 *M. oryzae* effector candidates and classified these based on temporal expression profile and structural conservation, laying the foundation for investigating the range of effector functions deployed by the fungus to cause rice blast disease.

## Materials and methods

### Fungal and plant growth conditions

Fungal (*M. oryzae*) strains and rice (*O. sativa*) plants were maintained as described previously (Talbot et al. 1993b). Ten-day-old plate cultures of *M. oryzae* were used to collect conidia for appressorium development assays, leaf sheath infections, and pathogenicity assays. Infected rice plants were incubated in a chamber at 24 °C, 12-h photoperiod with light intensity at 500 μmol m^−2^ s^−1^ using the metal halide bulb (Osram Powerstar HQI-Bt 400 w/d) and 90% relative humidity.

### Preparation of healthy and infected rice samples for RNA-seq analysis

*M. oryzae* conidia were harvested from 10-d-old complete medium plates (Oses-Ruiz et al. 2021). To carry out plant infections by spray assay, 21-d-old 3-leaf-stage rice plants were sprayed with conidia of Guy11 at 1 × 10^5^ spores mL^−1^ in 0.25% (w/v) gelatin. Infected rice leaves were harvested at 8-, 16-, 24-, 48-, 72-, 96-, and 144-h postinoculation. Rice leaves sprayed with 0.25% gelatin were used as controls to compare differentially expressed plant genes. To carry out plant infections by leaf drop infection, the third leaf of a 3-leaf-stage rice plant was placed on a flat surface and inoculated with a 20 µl suspension of 1 × 10^6^ spores mL^−1^ in 0.25% gelatin. A total of 20 lesions were harvested at each time point (8-, 16-, 24-, 48-, 72-, 96-, and 144-h postinoculation). Rice leaves inoculated with 0.25% gelatin were used in control experiments to compare differentially expressed plant genes. All collected samples were frozen in liquid nitrogen immediately for RNA extraction. Each experiment had 3 biological replicates (separate experiments).

### Total RNA purification and RNA sequencing

Total RNA was extracted from each sample using an RNeasy Plant Mini Kit (QIAGEN). An RNase-Free DNase Set (QIAGEN) was used to remove all genomic DNA. RNA quantity and integrity were measured on an Agilent 2100 Bioanalyzer, and purified RNA was used to make sequence libraries using a True-Seq RNA sample preparation kit from Illumina (Agilent). Sequencing was carried out using the HiSeq 2500 platform in standard mode.

### Differential gene expression analysis

Raw reads were separated by the Taxonomy ID of *M. oryzae* and *O. sativa* (NCBI: txid 318829 and txid 4530) using Kraken 2 (Wood et al. 2019). The extracted fungal reads from Kraken 2 were used to quantify the abundance of transcripts using Kallisto (Bray et al. 2016). TPM values were used to perform fungal mass estimation, PCA, and an overview of expression profiling analysis. Differential gene expression analysis was performed using Sleuth (Pimentel et al. 2017). Genes defined as upregulated had log2 fold change >1 and *P*-adjust < 0.05. Amino acid sequences of all coding genes from *M. oryzae* 70-15 were used to predict effectors. Venn diagram was generated using jvenn to compare different datasets (Bardou et al. 2014).

### Co-expression analysis and pathway enrichment analysis

WGCNA was used to analyze the gene co-expression network (Langfelder and Horvath 2008). Only genes with at least 5 fungal reads from all 3 replicates were considered. A total of 11,990 genes were, therefore, processed by WGCNA (version 1.69). The function BlockwiseModules was used to produce a network of a Pearson correlation matrix to examine the similarity between genes. A soft power threshold of 12 was chosen because it was the lowest power to obtain the lowest correlation value (0.85) from topology analysis (Supplemental Fig. S2B). Module detection was generated using modified settings to minimize the number of clusters using minModulesSize = 100, mergeCutHeight = 0.30. For each module, the expression level of the module eigengene was calculated to visualize co-expression patterns. For KEGG enrichment analysis, clusterProfiler (version 3.11) was used to perform Benjamini–Hochberg tests to obtain *P*-values and *q*-values (Yu et al. 2012). The enriched metabolic pathways were selected with *P* < 0.05.

### Construction of vectors and transformation of *M. oryzae*

All 32 selected effector candidates were cloned with their native promoters and entire protein-coding sequences without stop codons, then tagged at the C-terminus with GFP using recombination in vivo in yeast (*Saccharomyces cerevisiae*) (Oldenburg et al. 1997). In brief, the linearized pNEB-Nat-Yeast1284 cloning vector was used, which contains the *URA3* gene, allowing complementation of uracil (-) auxotrophy. The positive in-fusion plasmid was transformed into *Escherichia coli* to obtain plasmid DNA for fungal transformation (Supplemental Data Set S9 and S10). The promoters of the 17 selected *MEP* genes were further studied by tagging free cytoplasmic GFP using in-fusion cloning (Supplemental Data Sets S9–S11). To construct Mep1-GFP-NLS, primers TrpC-F-SpeI and TrpCp-R-EcoRI were used to amplify the TrpC promoter fragment from the hygromycin resistance gene cassette. Primers GFP-F-EcoRI and 3xNLS-R were used to obtain GFP-3xNLS from Addgene plasmid pEGFP-C1 EGFP-3xNLS. Primers TtrpC-nls-F and GFP-KpnI-R were used to amplify the TrpC terminator. All amplified fragments were inserted into linearized pCB1532 digested with SpeI and KpnI to generate TrpC-GFP-3xNLS-TtrpC. To generate Mep1-GFP-NLS, TrpC-GFP-3xNLS-TtrpC was digested with SpeI and EcoRI, and the in-fusion clone containing the Mep1 promoter and coding sequence was amplified with primers 353ProNLS-F and 353NLS-R from Guy11 genomic DNA. Gene deletions were generated by homologous recombination using the split-marker strategy or in-fusion cloning method. Confirmed positive vectors were transformed into protoplasts of the corresponding *M. oryzae* strains (Supplemental Data Sets S9–S11), as described previously (Talbot et al. 1993a).

### Live-cell imaging of *M. oryzae* during vegetative and invasive growth

To image conidia and mycelia of *M. oryzae*, samples were incubated on glass coverslips mounted with water. To image appressorium development, conidia were placed on inductive hydrophobic glass coverslips and the samples checked during appressorium development. To visualize disease progression on rice leaves, Wheat Germ Agglutinin-Alexa Fluor 488 conjugate (WGA-AF488, Thermo Fisher Scientific, catalog number W11261) was used to stain fungal hyphae, and PI (Merck Life Science, catalog number P4170) was used to stain the plant cell wall, as previously described (Redkar et al. 2018). To visualize the spatial localization of *M. oryzae* proteins of interest during plant infection, conidia were inoculated onto 3- to 4-wk-old leaf sheath samples of rice cultivar CO39 and infections were allowed to proceed for 0 to 48 h. Infected leaf sheaths were trimmed before microscopy (Kankanala et al. 2007). Confocal microscopy was carried out using a Leica SP8 laser confocal microscope. Excitation/emission wavelengths were 488/500–530 nm for eGFP, and 561/590–640 nm for mCherry. Images were analyzed using Leica software and ImageJ. An illustration of key stages during rice blast disease development in Fig. 2B was created with BioRender (https://biorender.com/).

### Statistical analyses

Statistical analyses were performed using the software Prism8 GraphPad. Data sets were tested for normal distribution before comparison. Data were analyzed using unpaired 2-tailed Student's *t*-test with Welch's correction when the data sets were normally distributed. When the data sets were non-normally distributed, the non-parametric Mann–Whitney test was used for comparisons.

### Accession numbers

All transcriptional profiling data are available through The European Nucleotide Archive (ENA) Accession Number PRJEB45007.

## Supplemental data

The following materials are available in the online version of this article.

**Supplemental Figure S1.** Assessment of the RNA-Seq Data Set of *M. oryzae* during rice infection.

**Supplemental Figure S2.** Sample clustering and the soft power analysis for WGCNA analysis.

**Supplemental Figure S3.** Biological process enrichment analysis of *M. oryzae* genes differentially expressed during plant infection.

**Supplemental Figure S4.** Hierarchical clustering of the expression of *M. oryzae* genes predicted to encode polyketide synthase during plant infection.

**Supplemental Figure S5.** Hierarchical clustering of *M. oryzae* genes predicted to encode fatty acid synthases during plant infection.

**Supplemental Figure S6.** Hierarchical clustering of *M. oryzae* genes predicted to encode cytochrome P450 mono-oxygenases during plant infection.

**Supplemental Figure S7.** Hierarchical clustering of *M. oryzae* genes predicted to encode ABC transporters during plant infection.

**Supplemental Figure S8.** Hierarchical clustering of *M. oryzae* genes predicted to encode transcription factors during plant infection.

**Supplemental Figure S9.** Visualization of the distribution of 863 *MEP* gene loci on the 7 chromosomes of *M. oryzae*.

**Supplemental Figure S10.** Cytoplasmically targeted Meps consistently localize to the BIC during biotrophic invasive growth of *M. oryzae*.

**Supplemental Figure S11.** Live-cell imaging of the secreted and non-secreted Mep1 protein variants during plant infection.

**Supplemental Figure S12.** Assessment of the expression of Mep effector candidates during plant infection.

**Supplemental Figure S13.** Targeted *MEP1* gene deletion in the rice blast fungus *M. oryzae*.

**Supplemental Figure S14.** Quantification of conidiogenesis from the *Δmep1* mutant.

**Supplemental Data Set S1.** Summary of read counts in this study.

**Supplemental Data Set S2.** Gene list of members of each WGCNA co-expression module during rice blast disease development.

**Supplemental Data Set S3.** Expression profile of predicted secreted protein-encoding genes differentially expressed during spray infection of rice cultivar CO39 by *M. oryzae*.

**Supplemental Data Set S4.** Expression profile of predicted secreted protein-encoding genes differentially expressed during spray infection of rice cultivar Moukoto by *M. oryzae*.

**Supplemental Data Set S5.** Expression profile of differentially expressed signal peptide-containing genes of *M. oryzae* during leaf drop infection of rice cultivar CO39.

**Supplemental Data Set S6.** List of differentially expressed *MEP* genes that are dependent on the Pmk1 MAP kinase for the differential expression during plant infection.

**Supplemental Data Set S7.** Expression modules and computationally predicted structural clusters of *M. oryzae* secreted proteins.

**Supplemental Data Set S8.** Summary of *M. oryzae* effector candidates characterized in this study.

**Supplemental Data Set S9.** Oligonucleotide primers used in this study.

**Supplemental Data Set S10.** Plasmids used in this study.

**Supplemental Data Set S11.** *M. oryzae* strains generated in this study.

**Supplemental Movie 1.** 3D visualization of plant infection at 16-h postinoculation.

**Supplemental Movie 2.** 3D visualization of plant infection at 24-h postinoculation.

**Supplemental Movie 3.** 3D visualization of plant infection at 48-h postinoculation.

**Supplemental Movie 4.** 3D visualization of plant infection at 72-h postinoculation.

**Supplemental Movie 5.** 3D visualization of plant infection at 96-h postinoculation.

**Supplemental Movie 6.** 3D visualization of plant infection at 144-h postinoculation.

**Supplemental Movie 7.** 3D live-cell imaging to show co-localization of Mep1^19–74^-GFP and Mep1-mCherry fluorescence during plant infection.

## Supplementary Material

koad036_Supplementary_Data

## Data Availability

RNA-seq data sets generated in this study have been deposited in the European Nucleotide Archive under the accession number provided. All other study data are included in the article and/or supporting information. All strains and plasmids generated in this study are available from the authors upon request.
