# Supplementary material for: The transcriptional landscape of plant infection by the rice blast fungus Magnaporthe oryzae reveals distinct families of temporally co-regulated and structurally conserved effectors
Source: Plant Cell. 2023 Feb 18;35(5):1360–85. doi: 10.1093/plcell/koad036 (PMC10118281; doi:10.1093/plcell/koad036)
Supplement: koad036_Supplementary_Data [file koad036_supplementary_data.zip › Supplemental Figures 114.pdf]

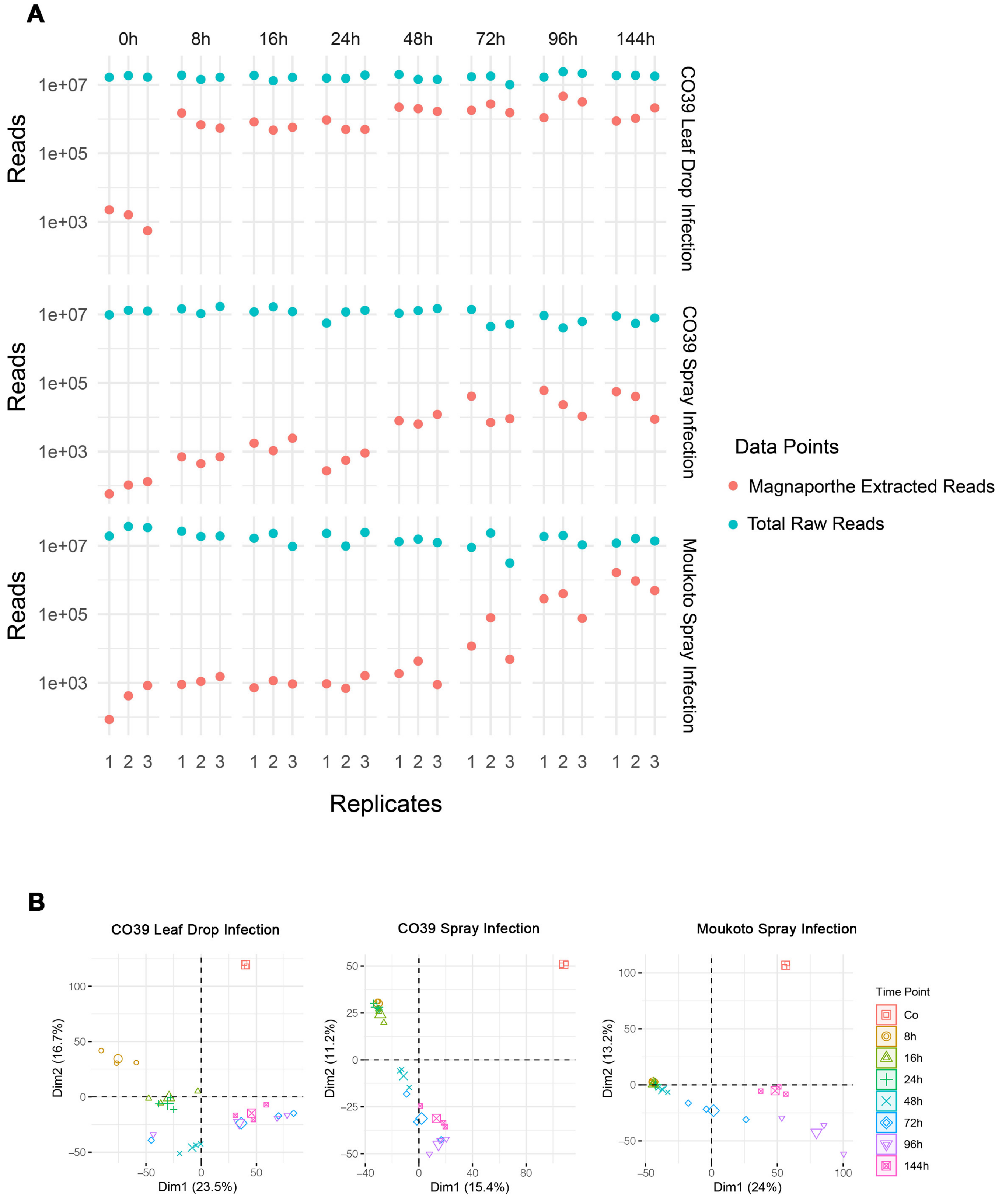

Supplemental Figure S1. Assessment of the RNA-Seq Data Set of *M.oryzae* during rice infection. (Supports Figure 1).
(A) Graph showing comparison of total raw reads and *M. oryzae* extracted reads from each inoculation method and cultivar-strain combination. **(B)** Principal Component Analysis (PCA) of fungal reads from the three infection datasets; CO39 Leaf Drop Infection, CO39 Spray Infection and Moukoto Spray Infection. Three independent biological replicates were generated for each time point 0h, 8h 16h, 24h, 48h, 72h, 96h and 144h.

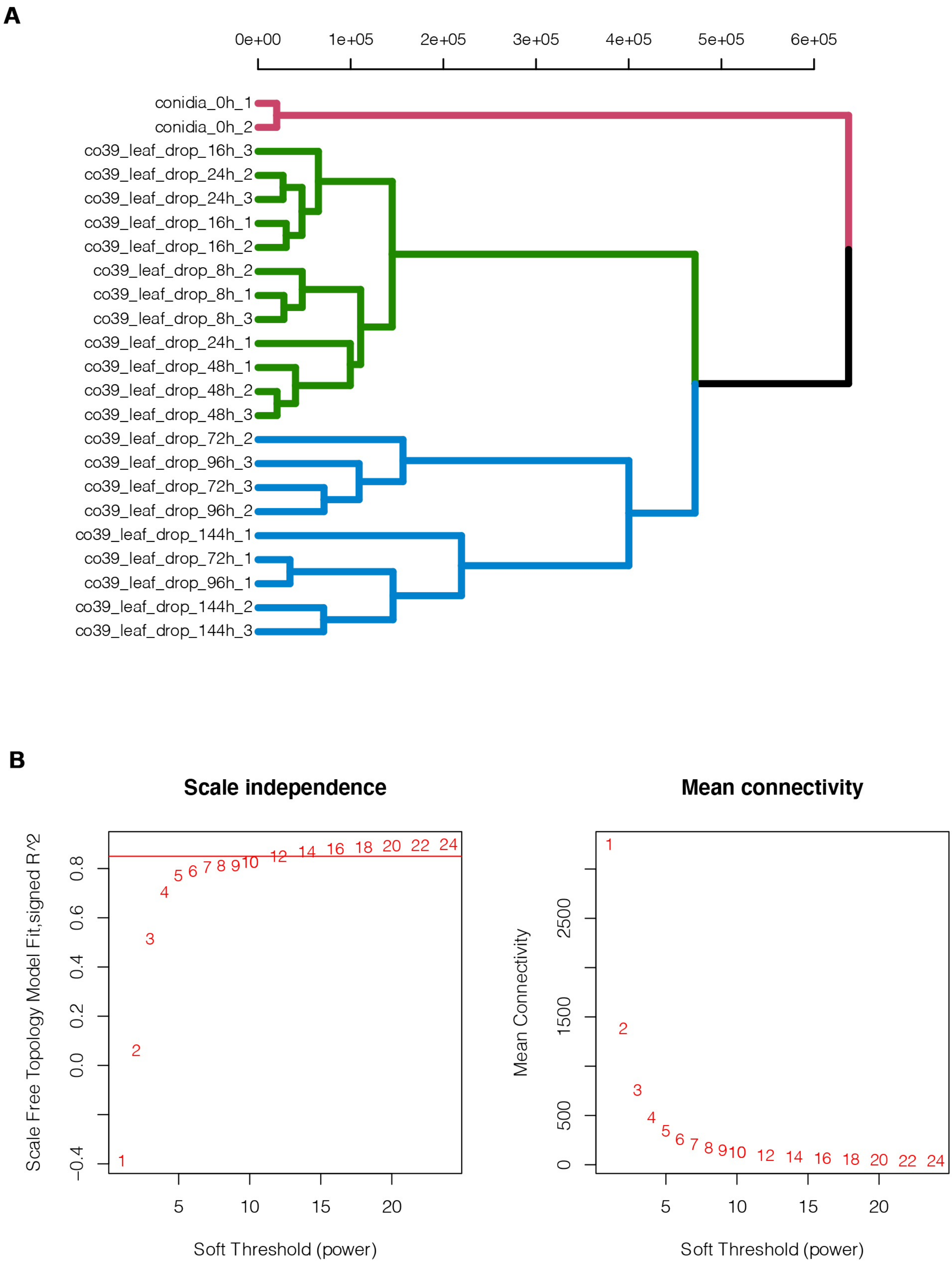

Supplemental Figure S2. Sample clustering and the soft power analysis for WGCNA analysis. (Supports Figure 2).
(A) Sample cluster analysis of all infected rice RNA-seq data sets. Conidia RNA-seq data sets are significantly different to those from the infection time course (shown in red). RNA-Seq Data Sets from early time points of infection, 8h, 16h, 24h and 48h, all clustered into a single clade (shown in green) while later time points from 72h, 96h and 144h grouped together into a third clade (shown in blue). **(B)** Soft power analysis to obtain the optimised power number for WGCNA analysis. The cut-off threshold indicated that the total RNA-Seq Data Set from infected rice samples could be divided into 10 co-expression modules.

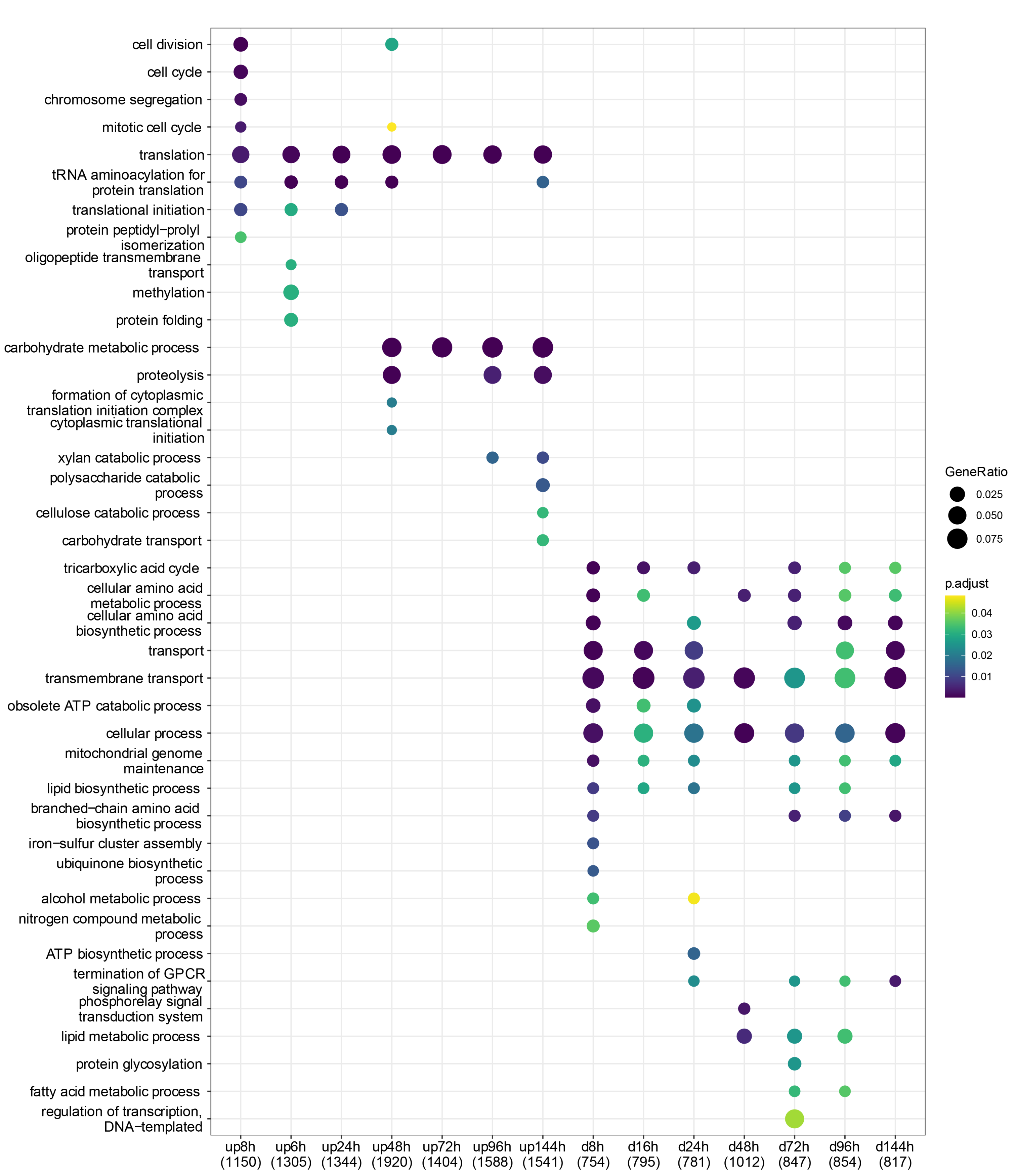
cell division
cell cycle
chromosome segregation
mitotic cell cycle
translation
tRNA aminoacylation for protein translation
translational initiation
protein peptidyl−prolyl isomerization
oligopeptide transmembrane transport
methylation
protein folding
carbohydrate metabolic process
proteolysis
formation of cytoplasmic translation initiation complex
cytoplasmic translational initiation
xylan catabolic process
polysaccharide catabolic process
cellulose catabolic process
carbohydrate transport
tricarboxylic acid cycle
cellular amino acid metabolic process
cellular amino acid biosynthetic process
transport
transmembrane transport
obsolete ATP catabolic process
cellular process
mitochondrial genome maintenance
lipid biosynthetic process
branched−chain amino acid biosynthetic process
iron−sulfur cluster assembly
ubiquinone biosynthetic process
alcohol metabolic process
nitrogen compound metabolic process
ATP biosynthetic process
termination of GPCR signaling pathway
phosphorelay signal transduction system
lipid metabolic process
protein glycosylation
fatty acid metabolic process
regulation of transcription, DNA−templated
up8h (1150)
up6h (1305)
up24h (1344)
up48h (1920)
up72h (1404)
up96h (1588)
up144h (1541)
d8h (754)
d16h (795)
d24h (781)
d48h (1012)
d72h (847)
d96h (854)
d144h (817)
GeneRatio
0.025
0.050
0.075
p.adjust
0.04
0.03
0.02
0.01

Supplemental Figure S3. Biological process enrichment analysis of *M. oryzae* genes differentially expressed during plant infection. (Supports Figure 2).
M. oryzae differential gene expression during plant infection was evaluated by comparison to expression in conidial mRNA using Sleuth (Pimentel et al., 2017), identifying genes showing a log2 fold change > 1, and P-adj < 0.05. Diagram shows up-regulated gene functions (top and marked 'up' in legend) and down-regulated gene functions (bottom and marked 'd' in legend) during a time series of *M. oryzae* infection based on leaf drop inoculation of rice cultivar CO-39 by strain Guy11. GO enrichment analysis was carried out using the R package "mogo" (https://github.com/TeamMacLean/mogo).

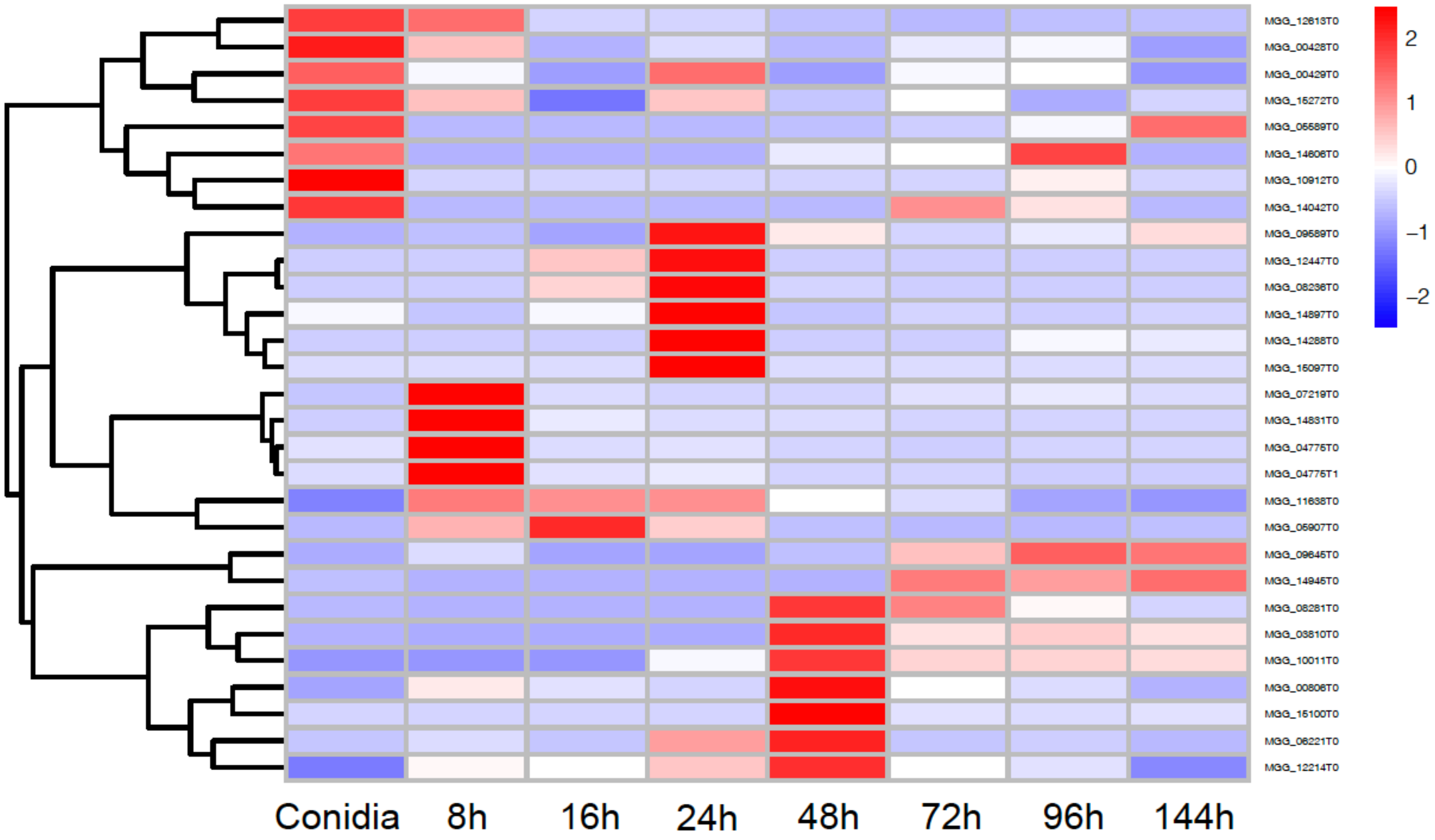

Supplemental Figure S4. Hierarchical clustering of the expression of *M. oryzae* genes predicted to encode polyketide synthase during plant infection. (Supports Figure 2).
Heat map showing the temporal pattern of the relative transcript abundance of 29 genes predicted to encode polyketide synthase during plant infection. Genome accession numbers are provided for each gene. Each gene is scaled to the mean TPM (transcript per kilobase million) value across all stages of infection-related-development and plant infection, with fold differences indicated according to the key.

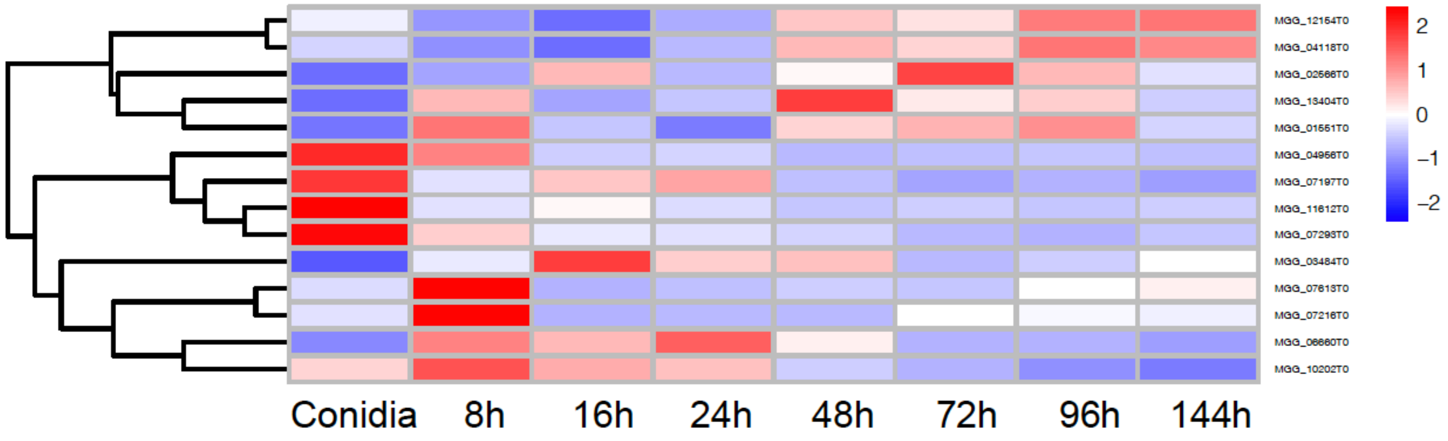

Supplemental Figure S5. Hierarchical clustering of *M. oryzae* genes predicted to encode fatty acid synthases during plant infection. (Supports Figure 2).
Heat map showing the temporal pattern of the relative transcript abundance of 14 genes predicted to encode fatty acid synthases during rice infection. TPM (transcript per kilobase million) value across all stages of infection-related-development and plant infection, with fold differences indicated according to the key.

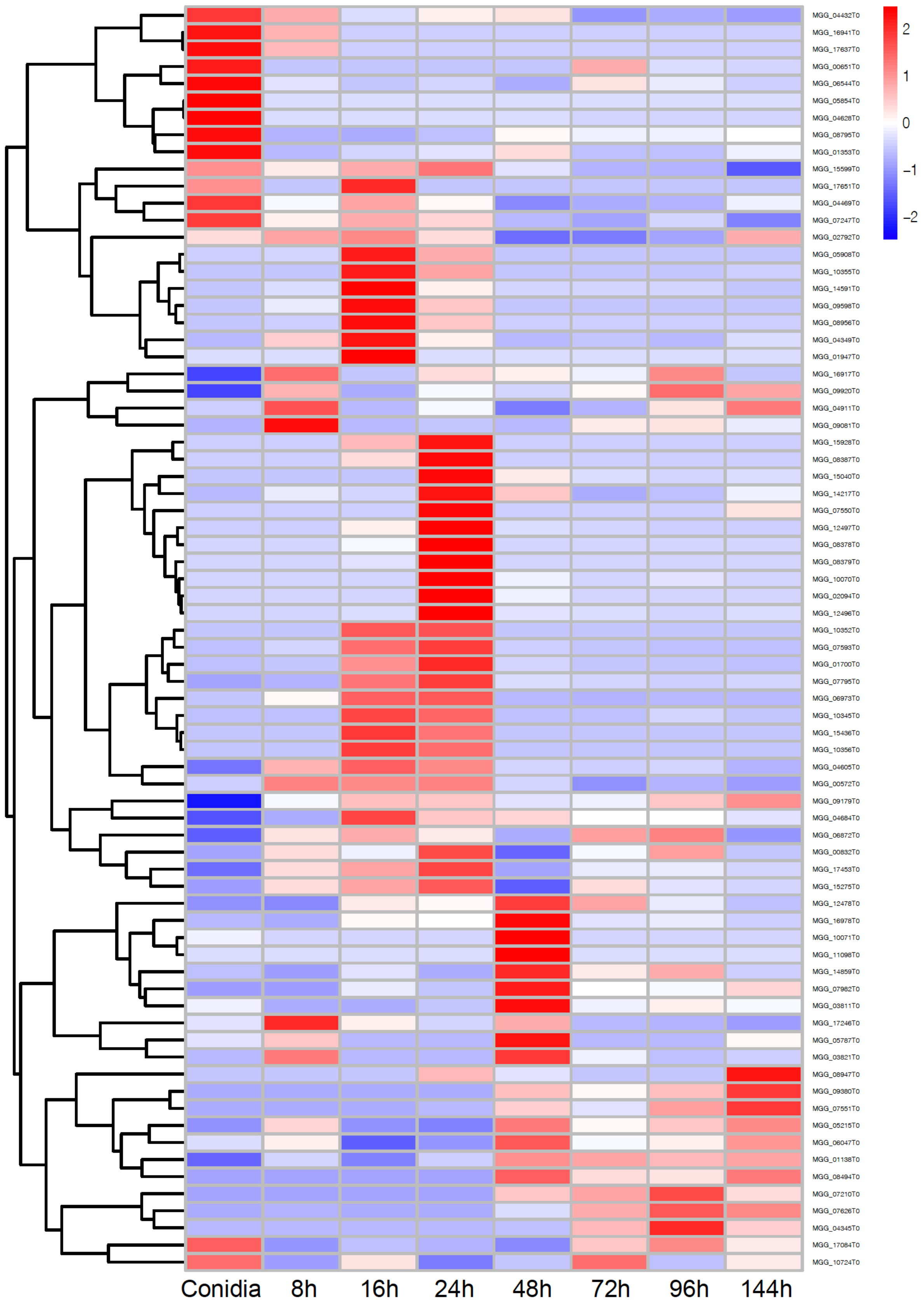

Supplemental Figure S6. Hierarchical clustering of *M. oryzae* genes predicted to encode cytochrome P450 mono-oxygenases during plant infection. (Supports Figure 2).
Heat map showing the temporal pattern of the relative transcript abundance of 75 genes encoding cytochrome P450 mono-oxygenases during rice infection. TPM (transcript per kilobase million) value across all stages of infection-related-development and plant infection, with fold differences indicated according to the key.

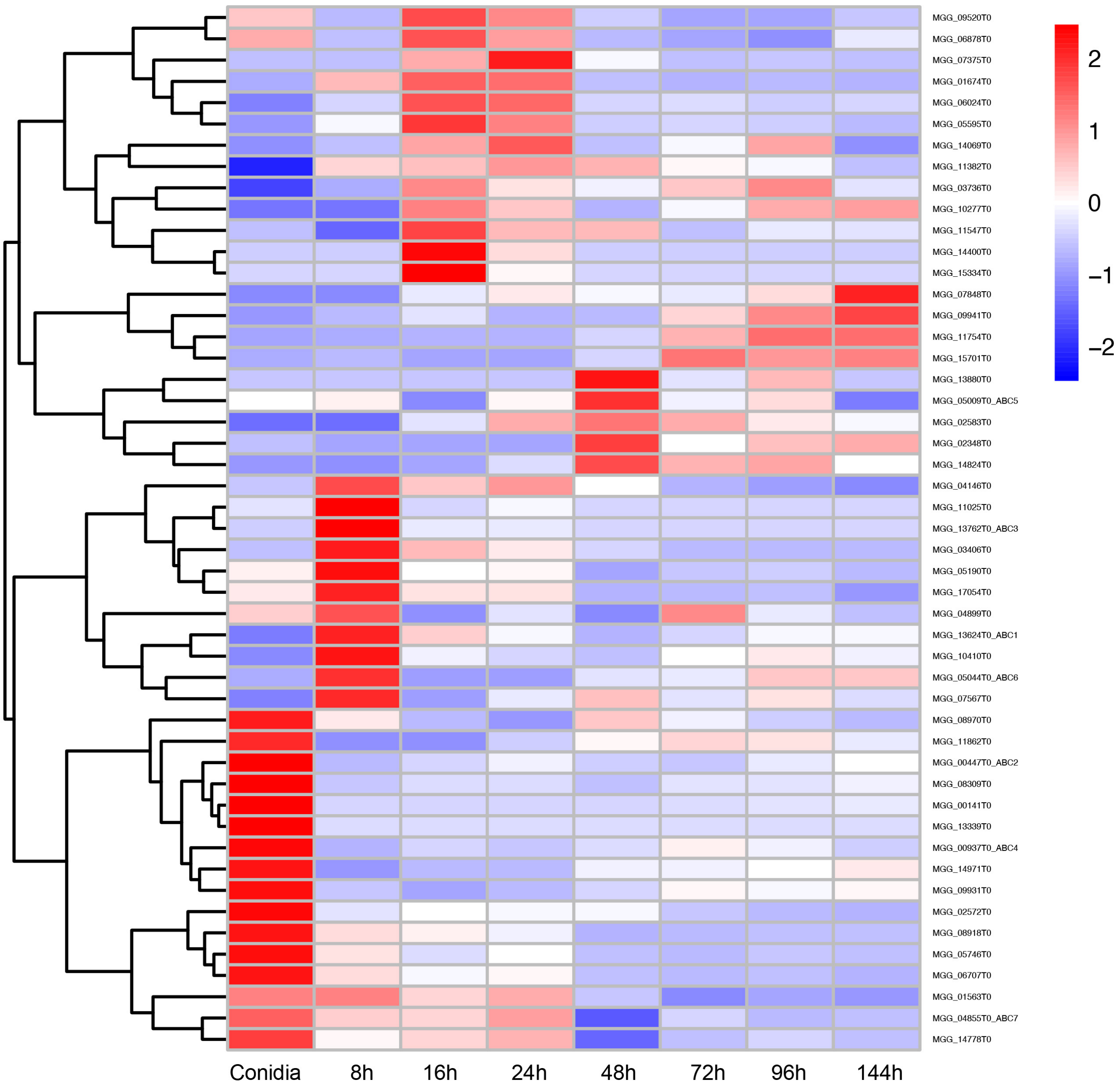

Supplemental Figure S7. Hierarchical clustering of *M. oryzae* genes predicted to encode ABC transporters during plant infection. (Supports Figure 2).
Heat map showing the temporal pattern of the relative transcript abundance of genes encoding ABC transporters. Candidate genes were extracted from the study of genome-scale analysis of ABC transporter genes (Kim et al., 2013). TPM (transcript per kilobase million) value across all stages of infection-related-development and plant infection, with fold differences indicated according to the key.

Supplemental Data. Yan et al. (2023). Plant Cell.

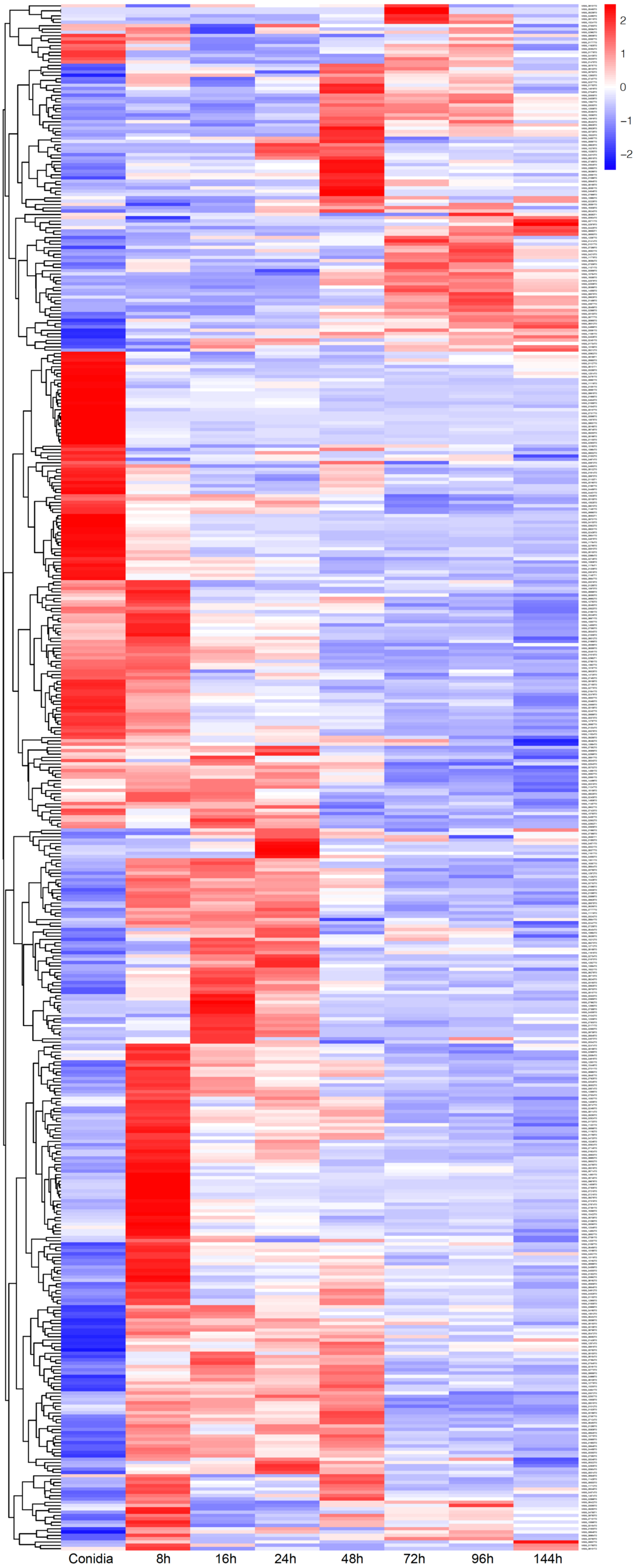

Supplemental Figure S8. Hierarchical clustering of *M. oryzae* genes predicted to encode transcription factors during plant infection. (Supports Figure 2).
Heat map showing the temporal pattern of the relative transcript abundance of 495 genes predicted to encode transcription factors during rice infection. All putative transcription factors were extracted from the Fungal Transcription Factor Database (http://ftfd.snu.ac.kr/magnaporthe), a platform designed to identify transcription factor-encoding genes in fungi. TPM (transcript per kilobase million) value across all stages of infection-related-development and plant infection, with fold differences indicated according to the key.

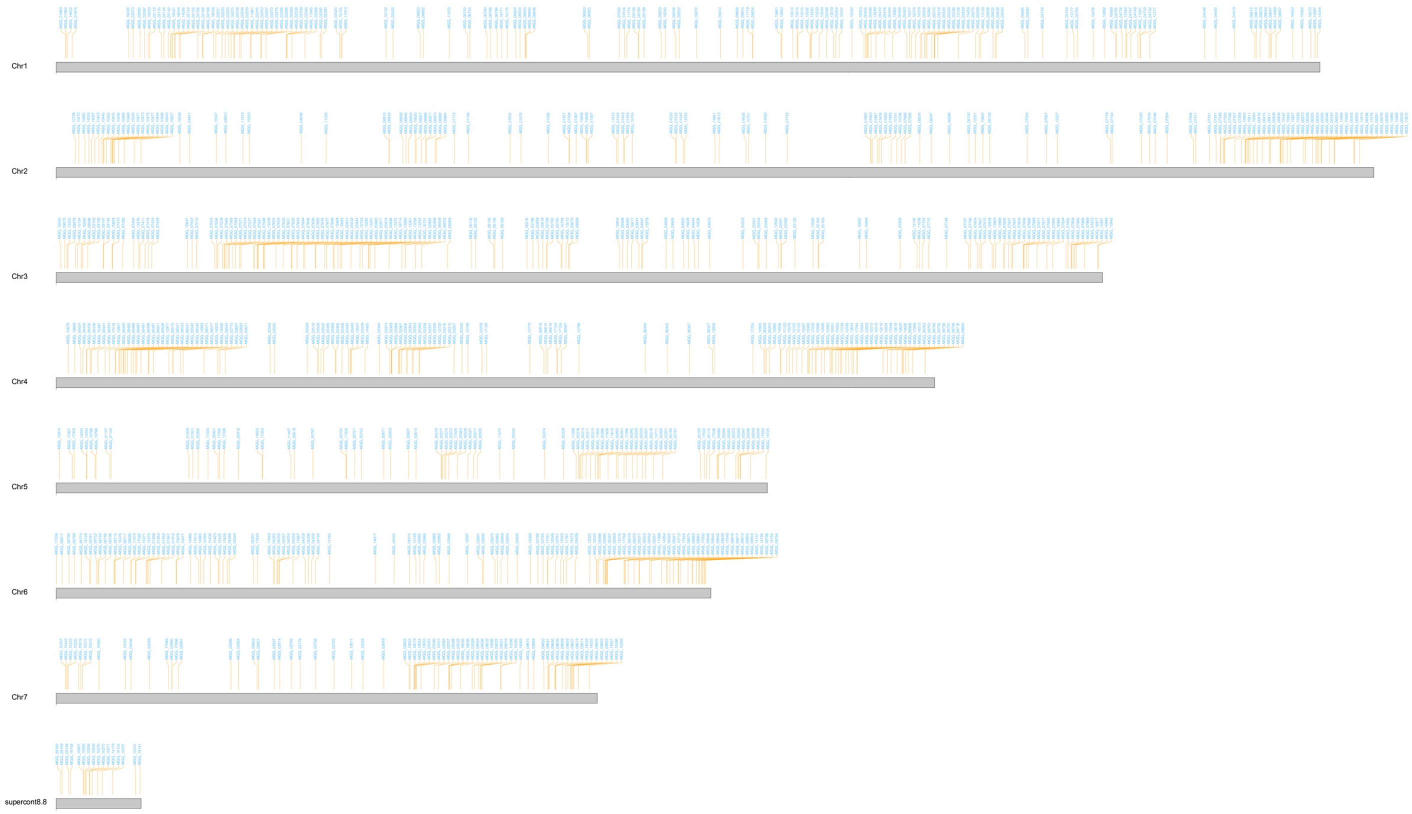

Supplemental Figure S9. Visualisation of the distribution of 863 *MEP* gene loci on the seven chromosomes of *M. oryzae*. (Supports Figure 3).

Genetic map showing the order and relative distribution of *MEP* loci among the seven chromosomes of *M. oryzae*. The positions were determined by analysis against the 70-15 reference genome (Dean et al., 2005).

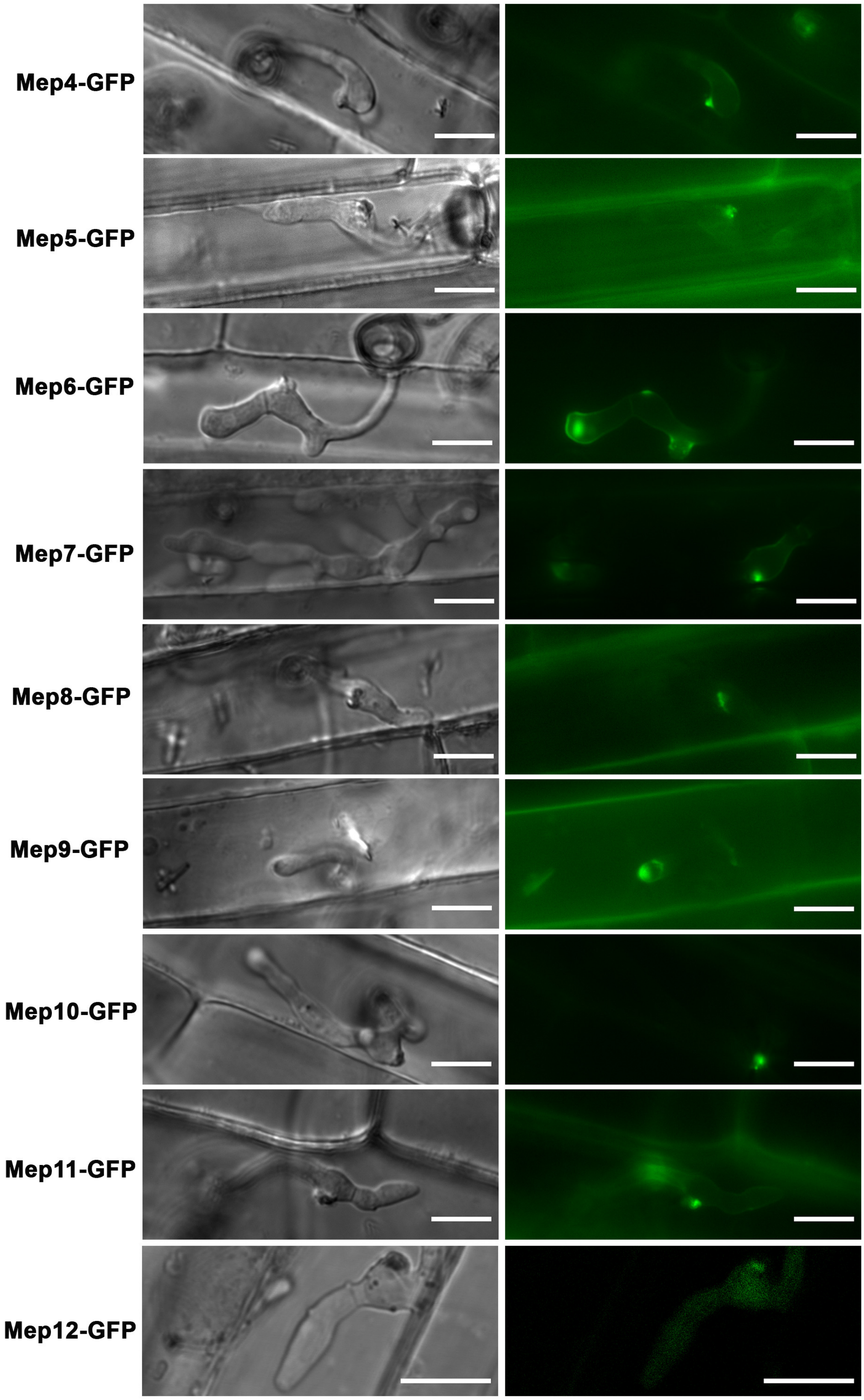

Supplemental Figure S10. Cytoplasmically targeted Meps consistently localise to the BIC during biotrophic invasive growth of *M. oryzae*. (Supports Figure 5).
Micrographs showing the localisation of Mep4, Mep5, Mep6, Mep7, Mep8, Mep9, Mep10, Mep11 and Mep12 which were all C-terminally tagged with GFP, transformed and expressed in Guy11 and used to carry out infections of rice leaf sheath of cultivar CO39. Laser confocal images were captured at 22-28 hpi. A single large fluorescent punctum, the BIC, was always observed in the initially invaded epidermal cell. Scale bars = 10 µm.

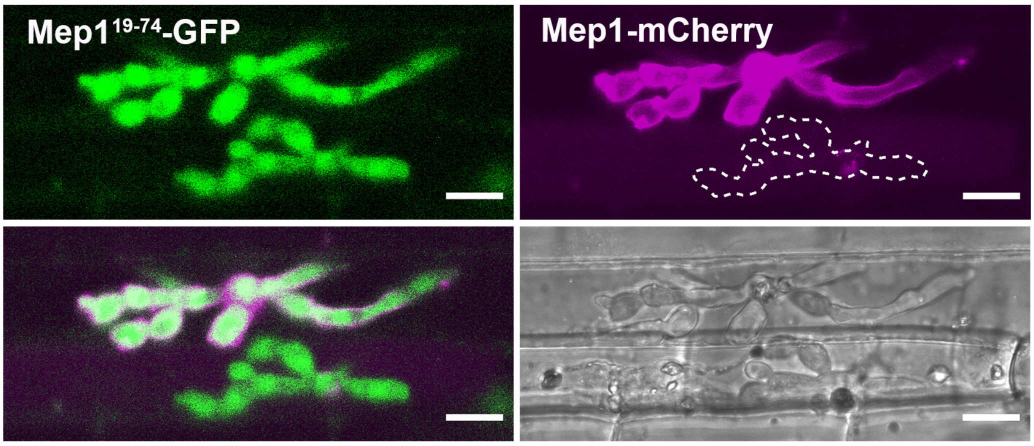

Supplemental Figure S11. Live-cell imaging of the secreted and non-secreted Mep1 protein variants during plant infection. (Supports Figure 6).
Micrographs showing invasive hyphae at two infection sites of *M. oryzae* Guy11 expressing Mep1$^{19-74}$-GFP and Mep1-mCherry. Two infection sites are present and can be observed by the GFP fluorescence signal from Mep1$^{19-74}$-GFP and by observation in the bright field channel. At one infection site, the green fluorescence from Mep1$^{19-74}$-GFP is uniformly enveloped by the magenta fluorescence from Mep1-mCherry. This is consistent with delivery of Mep1-mCherry into the apoplast between the fungal cell wall and extra-invasive hyphal membrane (EIHM). At the second infection site no Mep1-mCherry fluorescence could be observed. This suggests breakdown of the EIHM in the second infected cell in which Mep1-mCherry leaks into the whole cell, which loses viability. Laser confocal images were taken at 24 hpi. Scale bars = 10 um.

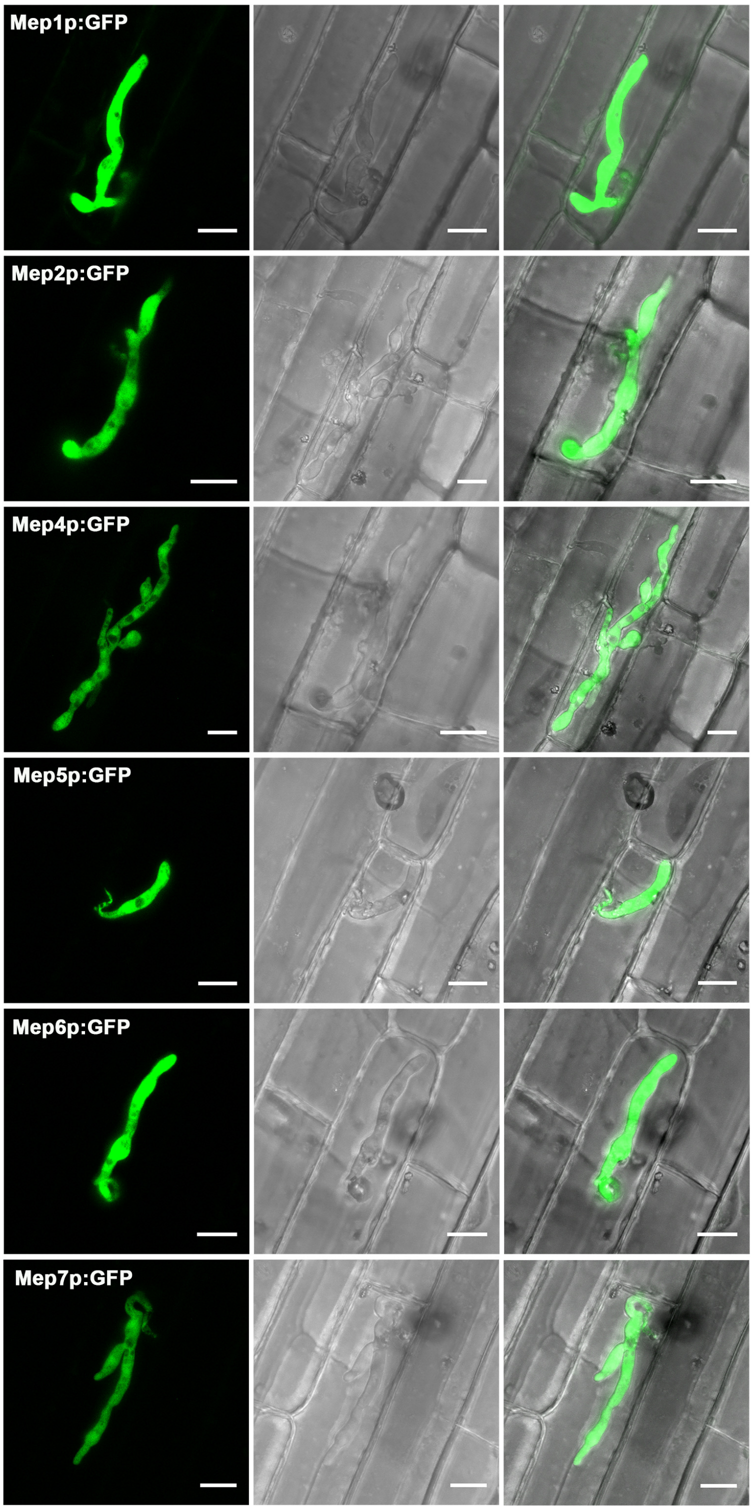

Supplemental Figure S12. Assessment of the expression of Mep effector candidates during plant infection. (Supports Figure 6).
Micrographs showing the expression of cytoplasmic GFP driven by the promoters of Mep effector candidates during plant infection. Promoters of *MEP1, MEP2, MEP4, MEP5, MEP6* and *MEP7* were fused to GFP, transformed into Guy11 and single copy transformants selected. A strong fluorescence signal was specifically observed inside invasive hyphae during plant infection. Scale bars = 10 µm.

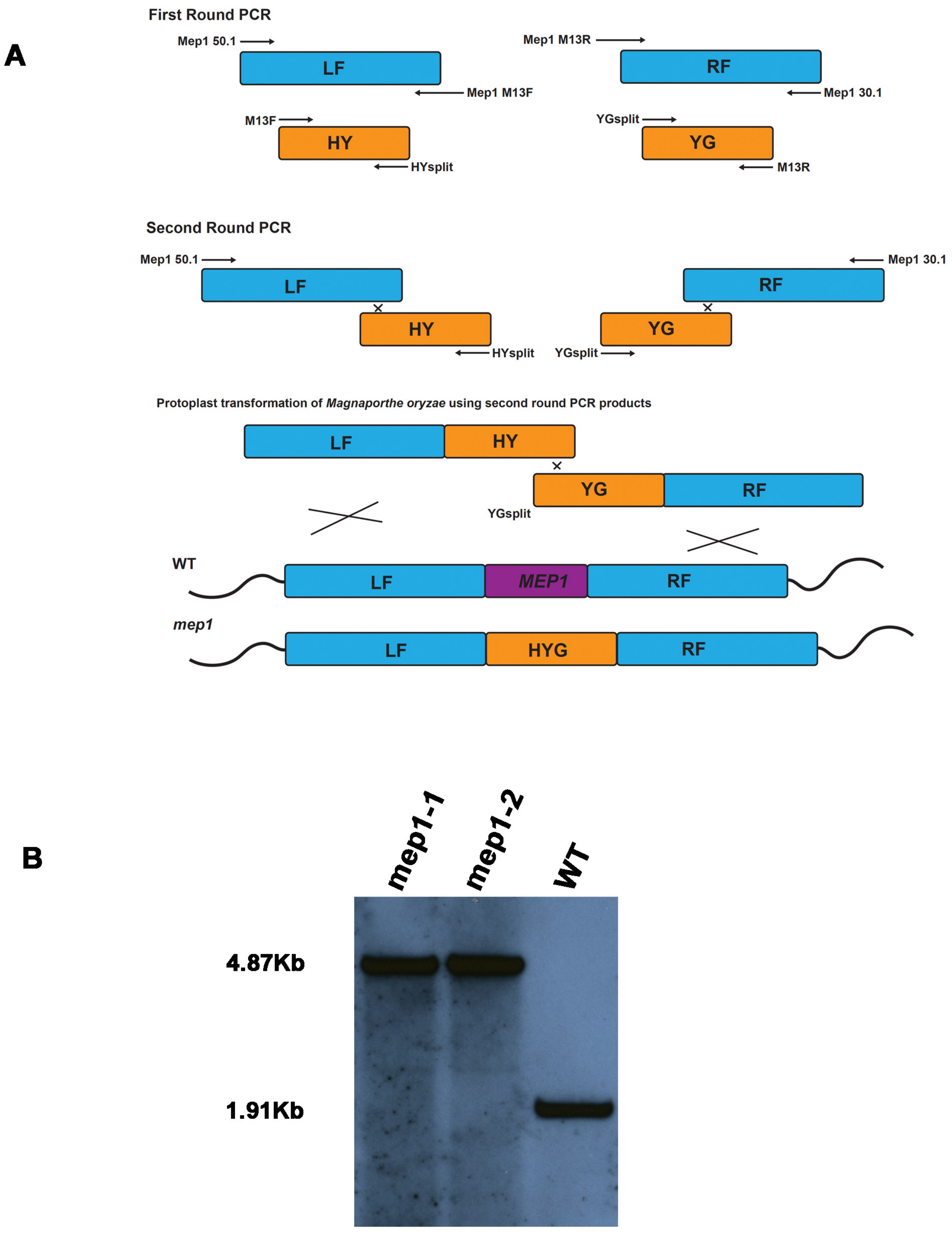

Supplemental Figure S13. Targeted *MEP1* gene deletion in the rice blast fungus *Magnaporthe oryzae*. (Supports Figure 9).
(A) Schematic representation of the *MEP1* gene deletion strategy using the split-marker strategy, as described in Methods. Two amplicons containing the left and right flanks of *MEP1* and each half of the hygromycin resistance gene cassette are used to transform Guy11. This results in three crossover events to generate a target gene replacement with re-synthesis of the whole hygromycin resistance cassette. **(B)** Southern blot to confirm targeted gene replacement of the *MEP1* gene. The same approach was used to generate gene replacement mutants for each *MEP* gene analysed in this study.

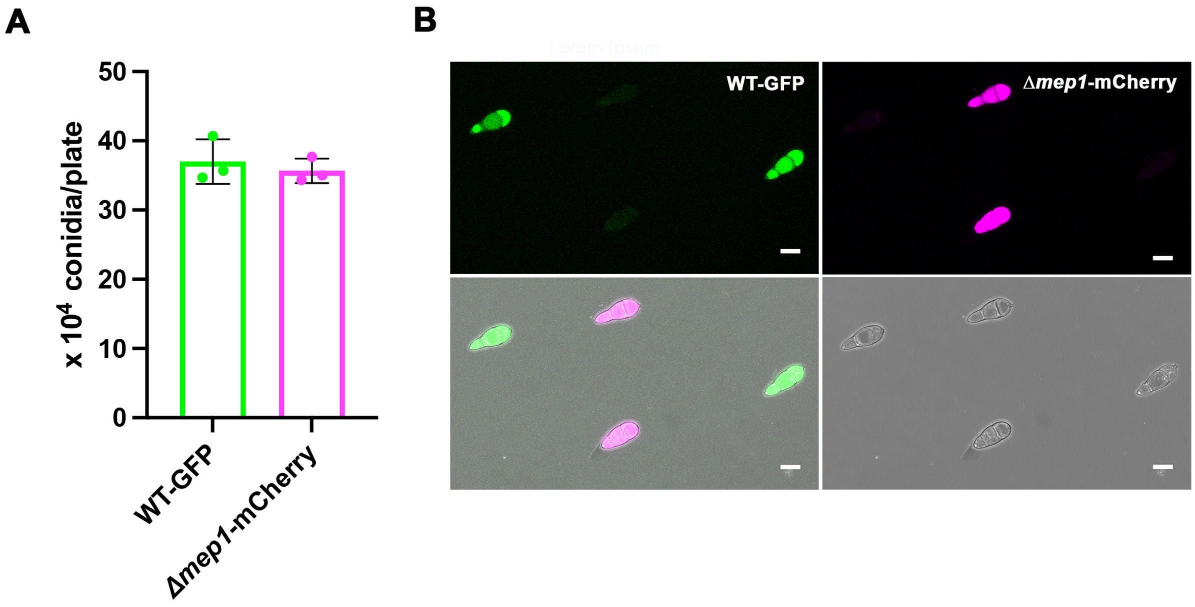

Supplemental Figure S14. Quantification of conidiogenesis from the Δ*mep1* mutant. (Supports Figure 9). In order to be able to use a mixed infection fitness assay, it was essential to ensure that each *M. oryzae* strain sporulated with the same frequency **(A)** Conidia were collected from WT-GFP and Δ*mep1*-mCherry and counted using a haemocytometer. Conidiation was analysed by quantifying conidia harvested from 10 day-old CM plate cultures of *M. oryzae*. Bar charts show the mean and standard deviation. No significant difference ($P > 0.05$) in conidiation was observed in three biological replicates of the experiment. **(B)** Laser confocal micrographs showing conidia of WT-GFP and Δ*mep1*-mCherry, respectively.
