# Supplementary material for: The transcriptional landscape of plant infection by the rice blast fungus Magnaporthe oryzae reveals distinct families of temporally co-regulated and structurally conserved effectors
Source: Plant Cell. 2023 Feb 18;35(5):1360–85. doi: 10.1093/plcell/koad036 (PMC10118281; doi:10.1093/plcell/koad036)
Supplement: koad036_Supplementary_Data [file koad036_supplementary_data.zip › Supplemental Movie legends.docx]

Supplemental Data. Yan et al. (2023). Plant Cell.

**Supplemental Movie Legends**

**Supplemental Movie 1.** 3D visualisation of plant infection at 16 hours post inoculation. (Supports Figure 1).

3D visualisation of rice cultivar CO39 leaves inoculated with *M. oryzae* Guy11 to show progression of tissue invasion. Infected rice leaves were collected at 16 hours post inoculation. Wheat Germ Agglutinin-Alexa Fluor 488 conjugate (WGA-AF488) was used to stain fungal hyphae and propidium iodide (PI) was used to stain the plant cell wall.

**Supplemental Movie 2.** 3D visualisation of plant infection at 24 hours post inoculation. (Supports Figure 1).

3D visualisation of rice cultivar CO39 leaves inoculated with *M. oryzae* Guy11 to show progression of tissue invasion. Infected rice leaves were collected at 24 hours post inoculation. Wheat Germ Agglutinin-Alexa Fluor 488 conjugate (WGA-AF488) was used to stain fungal hyphae and propidium iodide (PI) was used to stain the plant cell wall.

**Supplemental Movie 3.** 3D visualisation of plant infection at 48 hours post inoculation. (Supports Figure 1).

3D visualisation of rice cultivar CO39 leaves inoculated with *M. oryzae* Guy11 to show progression of tissue invasion. Infected rice leaves were collected at 48 hours post inoculation. Wheat Germ Agglutinin-Alexa Fluor 488 conjugate (WGA-AF488) was used to stain fungal hyphae and propidium iodide (PI) was used to stain the plant cell wall.

**Supplemental Movie 4.** 3D visualisation of plant infection at 72 hours post inoculation. (Supports Figure 1).

3D visualisation of rice cultivar CO39 leaves inoculated with *M. oryzae* Guy11 to show progression of tissue invasion. Infected rice leaves were collected at 72 hours post inoculation. Wheat Germ Agglutinin-Alexa Fluor 488 conjugate (WGA-AF488) was used to stain fungal hyphae and propidium iodide (PI) was used to stain the plant cell wall.

**Supplemental Movie 5.** 3D visualisation of plant infection at 96 hours post inoculation. (Supports Figure 1).

3D visualisation of rice cultivar CO39 leaves inoculated with *M. oryzae* Guy11 to show progression of tissue invasion. Infected rice leaves were collected at 96 hours post inoculation. Wheat Germ Agglutinin-Alexa Fluor 488 conjugate (WGA-AF488) was used to stain fungal hyphae and propidium iodide (PI) was used to stain the plant cell wall.

**Supplemental Movie 6.** 3D visualisation of plant infection at 144 hours post inoculation. (Supports Figure 1).

3D visualisation of rice cultivar CO39 leaves inoculated with *M. oryzae* Guy11 to show progression of tissue invasion. Infected rice leaves were collected at 144 hours post inoculation. Wheat Germ Agglutinin-Alexa Fluor 488 conjugate (WGA-AF488) was used to stain fungal hyphae and propidium iodide (PI) was used to stain the plant cell wall.

**Supplemental Movie 7.** Three dimensional live-cell imaging to show co-localization of Mep1^19-74^-GFP and Mep1-mCherry fluorescence during plant infection. (Supports Figure 6).

Movie showing a three-dimensional projection of invasive hyphae of *M. oryzae* expressing Mep1^19-74^-GFP and Mep1-mCherry growing within a living rice cell. Green fluorescence from GFP is uniformly enveloped by the magenta fluorescence from the secreted Mep1-mCherry. Laser confocal images were taken at 24 hpi. Scale bars = 10 um.
