# Supplementary material for: The transcriptional landscape of plant infection by the rice blast fungus Magnaporthe oryzae reveals distinct families of temporally co-regulated and structurally conserved effectors
Source: Plant Cell. 2023 Feb 18;35(5):1360–85. doi: 10.1093/plcell/koad036 (PMC10118281; doi:10.1093/plcell/koad036)
Supplement: koad036_Supplementary_Data [file tpc22.00671.PeerReviewReport.pdf › tpc22.00671.PeerReviewReport.pdf]

The transcriptional landscape of plant infection by the rice blast fungus *Magnaporthe oryzae* reveals distinct families of temporally co-regulated and structurally conserved effectors

Xia Yan, Bozeng Tang, Lauren S. Ryder, Dan MacLean, Vincent M. Were, Alice Bisola Eseola, Neftaly Cruz-Mireles, Weibin Ma, Andrew J. Foster, Miriam Osés-Ruiz[1] and Nicholas J. Talbot

Corresponding author: Nicholas J. Talbot, Nick.Talbot@tsl.ac.uk

Review timeline:

**TPC2022-LSB-00671**	Submission received:	July 13, 2022
	1st Decision:	Sept. 7, 2022 *revision requested*
**TPC2022-LSB-00671R1**	1st Revision received:	Nov. 21, 2022
	2nd Decision:	Dec. 21, 2022 *accept with minor revision*
**TPC2022-LSB-00671R2**	2nd Revision received:	Jan. 4, 2023
	3rd Decision:	Jan. 9, 2023 *acceptance pending, sent to science editor*
	Final acceptance:	Jan. 27, 2023

REPORT: (The report shows the major requests for revision and author responses. Minor comments for revision and miscellaneous correspondence are not included. The original format may not be reflected in this compilation, but the reviewer comments and author responses are not edited, except to correct minor typographical or spelling errors that could be a source of ambiguity.)

TPC2022-LSB-00671 1st Editorial decision – *revision requested* **Sept. 7, 2022**

We have received reviews of your manuscript entitled "The transcriptional landscape of plant infection by the rice blast fungus *Magnaporthe oryzae* reveals distinct families of temporally co-regulated and structurally conserved effectors." Thank you for submitting your best work to The Plant Cell. The editorial board agrees that the work you describe is substantive, falls within the scope of the journal, and may become acceptable for publication, pending revision and re-review.

Overall, the reviewers agreed that this time-course analysis of RNAseq datasets makes a valuable contribution to the MPMI research community. However, the reviewers also expressed several concerns that should be addressed either experimentally or editorially to clarify and strengthen the conclusions. In particular, please address the rationale for the choice of the 32 MEPs and explain the apparent inconsistencies in pathogen growth. In addition, the method used to evaluate the fitness of the mep1 mutant needs to include a control, such as WT-GFP vs WT-mCherry or WT vs another mep mutant (no fitness cost). Reviewer 2 also requested additional assays to confirm that rates of infection are similar between drop assays and leaf sheath assays. For example, one could assess whether representative genes from each module are expressed at the same time points in leaf sheaths.

-- Reviewer comments:

[Reviewer comments shown below along with author responses]

TPC2022-LSB-00671R1 1st Revision received **Nov. 21, 2022**

Reviewer comments and **author responses**:

Editor:

Overall, the reviewers agreed that this time-course analysis of RNAseq datasets makes a valuable contribution to the MPMI research community. However, the reviewers also expressed several concerns that should be addressed either experimentally or editorially to clarify and strengthen the conclusions. In particular, please address the rationale for the choice of the 32 MEPs and explain the apparent inconsistencies in pathogen growth. In addition, the

method used to evaluate the fitness of the mep1 mutant needs to include a control, such as WT-GFP vs WT-mCherry or WT vs another mep mutant (no fitness cost). Reviewer 2 also requested additional assays to confirm that rates of infection are similar between drop assays and leaf sheath assays. For example, one could assess whether representative genes from each module are expressed at the same time points in leaf sheaths.

We have focused our revision on responding to the points highlighted in the Editors' letter. In particular, we provide new Figures to show the extent of pathogen growth at each timepoint using whole leaf infections, which are identical to those used for the RNA-seq analysis presented. We therefore use only drop assay infections throughout, thereby precluding the need for comparisons with leaf sheath assays. We believe this deals with any potential confusion regarding stages of infection being analysed. We also provide control data as requested for the fitness assay using the isogenic wild type strain of Magnaporthe oryzae and also the complemented strain in which a wild type allele of *MEP1* is reintroduced in the the *mep1* mutant. We have also attempted to address as many of the other reviewer comments as possible.

Reviewer #1:

The infection processes of the rice blast fungus have been well characterized. In this study the authors generated RNA-seq data with 8 time points that are representative of different infection stages with two inoculation methods and two rice cultivars. Based on their expression files, M. oryzae genes expressed during plant infection were classified into 10 modules, which were found to be related to physiological and pathological shifts of the pathogen. Putative effectors (MEPs) were identified and analyzed for their chromosomal distributions, structural features, and expression patterns. Thirty-two MEPs were selected for functional characterization for their functions and localization during rice infection.

Although there are several publications on gene expression profiles of the blast fungus, this manuscript presented a much better/complete RNA-seq data of different blast infection stages and functionally. Besides functional characterization of 32 MEP genes, the authors also attempted to develop a method to assay the fitness of the mep1 mutant fitness, which is needed for the blast community.

We are grateful for these comments and positive evaluation of our study

My major concerns are:

1. The authors claimed that they 'functionally characterized 32 MEPs by live-cell imaging and targeted gene replacement' (line 372). As a 'large-scale biology' manuscript, data related to these 32 MEPs should be presented and described. For expression and localization, experimental data on Mep1 and Mep3 were presented. No data were presented on the mutants. Did the authors generate mutants for all these 32 MEPs? Also, it is not clear what were the criteria for selecting these 32 MEPs. If it is not randomly selected, general conclusions on MEP genes (such as 'Mep effectors are predominantly targeted to' -line 42), are not appropriate.

We now provide more information regarding the characterisation of the MEP gene selected, both in the manuscript, but also in Supplemental Data Set 8, which provides information on all experiments carried out.

We first investigated the localisation of all 32 Mep proteins by generating translational fusions to GFP expressed under their native promoters. These were each transformed into *M. oryzae* and single copy transformants selected. All were localised during infection by live cell imaging of the corresponding transformants. Only one apoplastic effector was identified (Mep1), with all others showing BIC localisation, consistent with them being cytoplasmic effectors. To confirm high level differential expression of MEP effector genes, we generated transcriptional promoter fusions of 17 *MEP* genes driven by their native promoters and showed that they were highly expressed during plant infection, consistent with the data shown for *MEP1* and *MEP3*.

We carried out targeted gene deletion for all 32 MEP genes, but in the case of *MEP4, MEP5*, *MEP8, MEP25* and *MEP28* we found that they were present in multiple copies in the *Magnaporthe* genome, therefore preventing selection of a targeted deletion mutant. Mutants of *MEP12, MEP20* and *MEP24* were independently reported during the course of our study (Kim et al., 2020 *Nat Commun* 11: 5845; Wu et al., 2015 *Chinese J Tropical Crops* 36:302-307) and *MEP11* encodes an allele of AvrPik which has been previously reported (Yoshida et al., 2009 *Plant Cell* 21: 1573-1591). We successfully generated targeted deletion mutants for the remaining 22 *MEP* genes (see Supplemental Data Set 8). The mutants do not show defects in growth and conidiation and do not have strong virulence

phenotypes. This was the motivation to establish a relative fitness assay in order to evaluate effector mutants in future.

The selection of the 32 *MEP* genes was essentially random. Fourteen *MEP* genes were selected as representative of the 63 *MEP* candidates identified in all infection experiments, 16 MEPs were expressed in both the CO39-leafdrop and Moukoto-spray infections, 1 candidate was only identified in the CO39-leafdrop and CO39-spray infections, and 1 candidate was only present in the CO39-leafdrop infections. We therefore selected the 32 genes to encompass as much of the variability in the MEP collection as possible. We now state clearly that thr selection was random.

2. To this reviewer, the RNA-seq data of an infection time course are impressive. Although the authors are interested in fungal genes, presenting some minimal analyses and descriptions on plant genes that may be correlated to the shifts in fungal gene expression will strengthen this Plant Cell manuscript.

We are grateful for the comment. In this manuscript we set out to focus on the transcriptional landscape of infection by the rice blast fungus and the consequent identification of termporally co-regulated effectors. The paper therefore focuses exclusively on fungal gene expression. Introducing an analysis of the landscape of transcriptional changes in the host would be beyond the scope of a single manuscript and would in our view reduce the focus of the study. All of the transcriptional profiling data is freely available so it can be analysed by anyone interested in host transcriptional responses. We will publish a full analysis and characterization of rice gene expression during infection in due course, so it can be a full and comprehensive analysis in its own right.

3. The authors should be commented on the effects to develop a method to test the fitness of the mep mutants. However, without a thorough characterization of the mep1 mutant, the results presented in this manuscript are not sufficient for their conclusions. Was the mep1 mutant defective in any stage of the infection cycle, including conidiation on infected plant leaves (lesions)? Quantitative assays with the virulence of the mep1 mutant also are necessary to show it has no defects in virulence. The authors may be able to use the GFP and mCherry to assay the biomasses of the wild type and mutants in co-infected leaves. Last, assaying with one MEP gene (MEP1) is not sufficient to reach general conclusions such as 'Mep effetcors contribute to pathogen fitness'-line 454.

The whole point of the fitness assay is that it enables very subtle differences in growth rate of mutants during plant infection to be compared. As the assay reveals, these differences may not be easily visible during a single round of infection but can be revealed by successive infections over three generations. We have now included additional control experiments to respond to these comments and those of the other reviewers (who all appreciated the novelty of the fitness assay) which are entirely consistent with our conclusions. The ∆*mep1* mutant does display reduced disease symptoms, so it does have a mild virulence phenotype, but the consequences of this mild phenotype are only revealed by carrying out the relative fitness assay. What this reveals is that even a minor defect in virulence (which may affect the rate of cell colonisation, lesion number/size and therefore overall conidial numbers) is sufficient to drive a strain carrying the mutation to extinction in a population over three generations. The other quantitative assays of virulence are not necessary. This is a new type of analysis for rice blast mutants and we feel it will have considerable value.

We agree that it is not possible to generalise about all effectors based on the reported data for *MEP1*, so we have modified the sentence accordingly.

Reword the sentence on line 306. ACE1 is not related to Nep1. And it may be better to describe ACE1 in M4 outside this part on secreted proteins.

Thank you. This has been reworded.

<u>Reviewer #2</u>:

Magnaporthe oryzae causes the devastating rice blast disease and also wheat blast, an emerging threat to wheat. Preventing rice blast would help secure global food security because despite deploying resistant rice cultivars and fungicides, 10 - 30 % of rice harvests are lost to this fungus. Although cellular and molecular studies have helped describe how the fungus invades rice and causes disease, a comprehensive study of the *M. oryzae* genes required for infection is lacking. This hinders the search for durable sources of resistance, not least because the full suite of *M. oryzae* effectors deployed to suppress host resistance is not known. Here, the authors tackle these open questions

by providing a detailed, temporal analysis of gene expression changes in *M. oryzae* from spore germination to symptom development.

The authors use high resolution RNAseq transcript analysis of two infected susceptible lines of rice, inoculated by two different methods, to show how *M. oryzae* genes can be classified into 10 modules of co-expressed transcripts corresponding to physiological changes during infection, including genes required for the production of secondary metabolites, and to the changing repertoire of secreted protein effectors. The work then falls into two general themes. In the first part, as a kind of means of calibrating the data, the authors show how genes and processes required for infection that have already been described in the literature can be identified in modules at the expected time points. The authors also identify fungal genes associated with secondary metabolism occurring during infection. In this manner, the authors show how, consistent with the literature, *M. oryzae* undergoes drastic changes in primary and secondary metabolism during invasive growth. In the second part of the paper, the authors focus on identifying likely secreted effectors, including a family of Max effectors with shared structural similarity, and determined how they are temporally co-regulated during plant infection. They then used this information to functionally validate a subset of "Mep" effector proteins to determine whether and how they are secreted. In this manner, the authors demonstrate how the analysis of co-regulated genes encoding likely secreted proteins might foster the discovery of previously unknown effector proteins. For one Mep gene, *MEP1*, the authors further show how this effector contributes to pathogen fitness in the host.

Some other *M. oryzae* RNAseq studies have been published previously. However, they focus either only on certain aspects of infection, for example appressorium morphogenesis, or cover only single time points. To my knowledge, this report describes the most ambitious and high-resolution *Magnaporthe* transcript profiling study to date. As such, it will be a great resource for the *Magnaporthe* community. Nonetheless, there are some areas of the study that require attention and/ or clarification:

We are grateful for this positive evaluation of our study.

Major Revisions

1. There is a problem with Figure 1A and the accompanying video. The left panel, showing rice cell colonization at 40 hpi, is correct and comparable to what has been described in other papers (eg Kankanala et al. 2007), namely that the fungus is filling up the first infected cell and starting to migrate to neighbouring cells. However, the right hand panel, at 48 hpi, is too overgrown for this time point. At 48 hpi, as correctly shown in the cartoon in Figure 2B, the fungus has filled the first infected cell and has sent several strands of invasive hyphae into neighbouring cells, but these have not progressed far into the neighbouring cells. As shown correctly in Fig 2B, migration into the third layer of cells and beyond is not evident until around 96 hpi to 144 hpi. Therefore, the right hand panel in Fig 1A is either an image taken at 96 hpi rather than the stated 48 hpi, or is significantly overgrown for reasons that are not evident. Although the figure legend states the rice line used was CO39, perhaps there was a mix-up and this is Moukoto? Either way, this issue needs to be resolved because if this is indeed an image of infection at 48 hpi as stated, and therefore massive overgrowth was observed by the authors, then this conceivably could render the modules either internally meaningless, or impossible to compare across research groups, many of whom observe infection proceeding at the rates shown in Fig 2B rather than Fig 1A. Note too that the whole leaf infection images in Figure 1B are blurry and the lesions for Guy11 in III are not typical and are less spreading than expected.

In order to remove the potential for confusion, we decided to replace all the panels in Figure 1A to show stages of rice infection from leaf drop inoculations of whole leaves of cultivar CO39. These now directly correspond to the material used in the RNA-seq analysis. 3D movies of each time point are also provided. As the reviewer correctly points out, there are often discrepancies in timing with leaf sheath assays, so we felt it best to not use these to illustrate stages of development, since the RNA-seq data did not use this material, but always used whole leaves.

The fungus was visualised by staining with WGA-AF488 and plant tissue with propidium iodide. The stage of development now do correspond with the schematic representations shown in Fig 2B. Our intention with Fig 2B was simply to be able to enable the reader to navigate the stages of infection overall. They are schematic representation only, but in association with the new Fig. 1A, it should be possible to readily determine the temporal dynamics of rice blast infections.

We also now provide higher resolution images for Figure 1B. The previous images were not provided at a sufficiently high resolution. We have used the same leaf images because these are from the actual infections from which RNA was extracted. The disease lesions for Guy11 in are those observed with spray infection of CO39 at this time point (Leaf III) under the conditions we used for infection assays. The lesions will spread further and coalesce at later time points. CO39 is a moderately susceptible rice cultivar and lesions are smaller than those observed for Moukoto infections.

2. The modules in Fig 2A are defined based on the cartoons in Fig 2B, which are themselves derived from previous studies from a number of groups using optically clear leaf sheath assays. However, the data underlying the modules are transcripts from whole leaf droplet assays and whole plant infections. Particularly regarding the leaf drop assay, which provides the most transcripts, I wonder whether this is a correct assumption. Did the authors show that the rates and timing of cell to cell movement, and the transition to necrotrophy, is conserved in whole leaf inoculations compared to leaf sheath assays? If not, then the modules in Fig 2A cannot be mapped onto the time course in Fig 2B.

We agree with this comment which is why we have provided the new images in Fig. 1A from whole rice leaves inoculated by droplet infection, thus representing the exact tissue type used for the RNA-seq data from which the co-expression modules were defined. The modules in Fig 2A are based on WGCNA of highly correlated co-expressed genes (Langfelder and Horvath, 2008, *BMC Bioinformatics* 9: 559). The schematic figures in Fig. 2B are therefore presented as a guide, but the definitive images in Fig. 1A and accompanying 3D movies are now provided too to prevent any ambiguity.

I am also confused by a statement at Line 168, where the authors claim that by using the highly susceptible Moukoto line, they show a greater number of *M. oryzae* sequence reads at earlier infection and throughout disease progression compared to CO39. However, this is only evident in Fig 1C at 96 hpi and 144 hpi. Can the authors explain what they mean by "throughout".

This was a confusing sentence as we were comparing the spray infection of Moukoto and CO-39, in which there are more reads observed at earlier time-points, although this is not clear from the bar chart. To avoid this potential confusion, we have only compared later stages of infection and altered the sentence to say "By utilizing the highly susceptible Moukoto, we were able to show a greater number of *M. oryzae* sequence reads at later stage of disease progression compared with CO39 infections, as shown in Figure 1C"

3. Transcripts are binned into 10 co-expression modules shown in Fig 2A. However, the error bars for some, eg module 4, 6, 8, 9 and particularly 10, are so large that there is a likelihood that no differences exist at the stated time points. Maybe I am missing something here, and I do not have working familiarity with eigengenes, but some of the claims for elevated transcripts at particular time points do not seem well supported and the notion of co-expressed modules is in jeopardy. I would at least expect to see statistics included in the panels in Fig 2A to show significant differences exist. Or, the authors might better need to explain why they conclude that genes peak at certain time, for example after 72 hpi in Module 10, when the graph of Module 10 in Fig 2A carries enormous error bars.

The reviewer makes a good point regarding the large range bars in the eigengene profile plots of Figure 2A. The reviewer points out that modules 4,6, 8, 9 and 10 may not be different at specific time points, based on the ranges of expression. We have not claimed that the average increase at the peak is tested to be specifically different from 0. This isn't the aim or a prerequisite of the clustering, which aims first to group the most similar genes together, and assess their expression generally. Briefly, the modules are formed by calculating a correlation coefficient between all genes which places the genes in a similarity space that we can visualise conceptually as spots in a 2D plane. The job of the clustering step in WGCNA is to draw lines around the tightest clouds of spots such that the most similar expression profiles considered across the entire timecourse at once are grouped together. When we do this, we can see that clear differences between the modules exist and it is clear that each module is a group of genes whose behaviour is different from all others because there are unique parts to each profile the reviewer questions that distinguish them. For example, if the variable peak of Module 4 were not present, and a value of around 0 were plot, that profile would not reasonably fit into any other module. For example, Module 4 genes lack the Co peak of M1, the 8h Peak of 2 and the sustained 48h – 96h peak of Module 6 etc. Each of the modules can be seen to be distinct in this way.

The number of clusters and correlation coefficients were determined robustly and conservatively according to the

best practices and thresholds of the WGCNA tool (Langfelder and Horvath, 2008 BMC Bioinformatics 9: 559) and to us it seems fair to interpret all these modules as distinct from each other in the correlation similarity space. For a gene to fall into a module it would need to be closer to that module than any other. The Modules with peaks with large range markers, while noisy, are nonetheless on average increasing at these points and our interpretation, eg for Module 10 that these are genes that are co-regulated and generally increased to a peak at 72h seems reasonable. Further evidence that these modules are biologically coherent comes from the ontology analysis in Figure 2C. All the modules show significant over-representation of biological function annotations with conservative p-values, which would not likely happen if the modules were incoherent. We have tried to make this clearer in the text in light of the concern raised.

Note that the error bar is missing at 72 hpi in Module 7.

This has now been corrected.

4. Assuming the 10 modules are correct and blocks of genes are co-regulated, including different suites of transcription factors, can the authors shed light on what controls these changes? If it is solely due to transcription factors, is it possible to perform network analysis to specifically link suites of gene expression in modules to co-expressed transcription factors? I felt more was needed to assess what might control the discrete changes in gene expression as disease progressed. Might there be a role here for chromatin remodeling? In my opinion, at least some discussion of how groups of genes might be temporally co-regulated is warranted.

We do not feel confident inferring specific transcriptional regulators in the co-regulation of genes during infection at this point, which is why we have not carried out the analyses suggested. We are addressing the identification of potential transcriptional regulators of effector gene expression using a forward genetics approach (see our *BioRxiv* submission Tang et al., 2022 https://www.biorxiv.org/content/10.1101/2022.09.04.506535v1.full) and then correlating the resulting mutant with the data sets presented in this manuscript. Recent published data does provide evidence that histone modification dynamics at H3K27 may play an important regulatory role in *M. oryzae* and this study is now cited (Zhang et al., 2021 *PLoS Genetics*17:e1009376).

5. The mixed infection assay to study the function of Mep1 is a novel and inspired approach. However, there could be other direct or indirect effects on fungal physiology that arise from the deletion of the *MEP1* gene. For example, merely going through the transformation process might reduce fitness in the Δ*mep1* strain. One way to assess this particular concern would be repeat the experiment with a complementation strain whereby the Δ*mep1* mutant also carries a functional copy of *MEP1*. If the loss of fitness in Δ*mep1* is solely due to the loss of Mep1, then we would predict that the complementation strain is not reduced in fitness compared to Guy11.

We agree with the reviewer comment. We have carried out the control experiments suggested by using two wild type strains, transformed with the relevant markers, which did not show any fitness defect associated with transformation. We also carried out infection using a complemented Δ*mep1* mutant carrying a functional copy of *MEP1.* We observed complementation of the fitness phenotype as shown in Figure 9B.

Another means to assess the likelihood that an effector has a physiologically meaningful role on the plant host is to transiently express it in *Nicotiana benthamiana* and assess whether it triggers cell death. Can Mep1 be tested *in planta* in such a manner?

We aim to present a full functional analysis of the Mep1 effector in due course, but this is beyond the scope of this paper, which describes the entire Mep effector data set. We have transiently expressed Mep1 in *Nicotiana benthamiana* and it does not trigger cell death, so including this information would not add useful information to the study. We are carrying out additional lines of investigation, including co-immuno-precipitation-mass spectrometry analysis in order to determine the function of Mep1.

6. The sentence at line 549 states "During early biotrophic colonisation of leaf tissue significantly up-regulated genes associated with carbon and nitrogen source acquisition from the plant host by 24h." However, not only is this sentence a little garbled or truncated, but at least in the main text, no transporter genes were noted. Nutrient acquisition from the host is an important but poorly understood process and at a minimum, a few examples of specific transporter genes upregulated in the modules during infection should be discussed.

We agree with the reviewer comment. We have modified the sentence for clarity. We have now also provided

examples of transporter genes that are up-regulated during infection and present a heatmap of transporter-associated gene functions in Supplemental Figure 7.

Minor Revisions

1. There is a callout for "Supplemental Figure X" in line 333, please correct this.

This has now been corrected.

2. In the section on stage-specific expression of Mep effectors, it is not clear why 63 *MEP* genes identified from all the experiments were selected, and then why only 32 of those were functionally characterized. What is the rational for the selecting the 63 and further analysing the 32?

From the set of 63 *MEP* genes identified in all RNA-seq analysis experiments, 23 of them have been reported previously including Avr-Pita1, PWL2, Bas1, Bas3 and Bas4. We selected the 32 *MEP* genes randomly, as detailed in response to Reviewer 1. They were selected to encompass as much of the variability in the *MEP* collection as possible, but the selection is essentially random.

3. As far as I can tell, supplemental movie 2 is missing.

Thank you for making us aware of this. It has now been re-uploaded.

4. In Fig 5C, Mep2 is shown in the BIC but it is not mentioned in the text. Is this a typo or was it used as a control?

Mep2 is one of the 32 characterized Meps. It is in the structure cluster 7 (Figure 4). It is simply shown to provide a second example of BIC-localised effector. A further 9 examples are shown in Supplemental Figure 10.

5. On line 390, Supplemental Figure 5 is called out, but this should be Supplemental Figure 9, please correct.

This has been corrected.

6. Supplemental Figure 11 is called out in the text before Supplemental Figure 10. Can these figures be renumbered?

This has been renumbered.

7. In my opinion, the sentence "physiological processes and genes involved in secondary metabolism expressed...." should be replaced with the more accurate "physiological processes and genes involved in primary and secondary metabolism expressed...."

The sentence has been rephrased as suggested.

Reviewer #3:

This submission by Yan et al describes a comprehensive analysis of the infection transcriptome of *M. oryzae* over time. It provides a good insight into total gene regulation control and highlights that repression of gene expression is as important as upregulation during developmental transitions. It illustrates the metabolic reprogramming that occurs during different phases of the life cycle. The dataset is a valuable resource for the community and the analyses by the authors provide interesting and fundamental insights into the infection process and the nature of *M. oryzae* effectors. The clustering of certain structural features and expression patterns is particularly interesting and should stimulate similar analyses for the potential effector repertoires of other plant pathogens.

We are grateful for the positive evaluation of our study.

I only have fairly minor questions and comments.

What was the rationale of only doing the drop inoculations on the CO39 leaves and not also on Moukoto? Please include an explanation.

We decided to use the Guy11-CO39 leaf drop infection assay as the main source of data, given the wide usage of this strain-cultivar combination in the rice blast research community globally. We felt it would be of the widest possibly utility. We used Moukoto infections as an example of a highly susceptible host. We did not feel it necessary to carry out leaf drop infections of Moukoto, but may do this in future. We felt that the data set provided

here would be likely to be most applicable to studies across the world, which was our prime motivation in creating the resource.

Do the different inoculation modes reveal anything other than that the droplet inoculation gives more infection and thus a higher number of reads representing the pathogen? Do the authors think that the 20 (21?) effectors only revealed in spray inoculations (Fig3) reveals something significant? (Could it be linked to a lower density of inoculum?) Please discuss.

This is an interesting point. The main difference was, indeed, that droplet infections enable more sequence reads from the pathogen to be generated because of a high maount of infection in a small region of the leaf tissue. However, there were other differences observed, as pointed out by the reviewer and shown in Figure 3, such as effector candidates only onserve in spray infections. We ensured some examples of such MEP effectors in the 32 selected for further study. However, we do feel that there should be a complete study of the 21 differentially expressed genes only present in the spray inoculations to see if there are density-dependent regulatory processes. This is, however, a major study in its own right and we do not feel it could be included in this study.

Line 166: Regarding the classification of reads: did the authors cross-compare and discard reads that had hits with both rice and fungus (and were thus unclassifiable)?

We used the software package Kraken 2, which is a taxonomic classification system, to classify reads. Briefly, this uses a single database of many genomes, including the rice and Magnaporthe genomes, and evaluates k-mers to find the taxa that have the highest number of kmers in common with the generated reads. A given read is then classified into specific taxa. There is therefore no concept of a read being 'a hit to rice and fungus', but reads that contain k-mer sets that were not classified explicitly as rice or *Magnaporthe oryza*e reads were not used further.

Fig2c: The legend does not make it clear as to what exactly is represented by the spots. What is the definition of an over-represented physiological function (i.e. compared to what)?

The KEGG over-representation analysis is a statistical analysis of genes that are classified as encoding products within specific KEGG pathways that are also over-represented in each WGCNA expression module. The color of the spot indicates a corresponding P-value, as shown in the Figure. The size of the spot indicates the GeneRatio, which is the number of genes in the tested KEGG pathway compared to the total number of genes detected with KEGG pathway in each module. The larger the spot, the more genes from the given physiological process/pathway. The enriched pathways were selected with a P-value < 0.05 as stated in Material and Methods.

Fig 3C legend: 'DEG sec' is not used in this fig nor elsewhere, change to 'DEG secreted' as in the figure label.

This has now been changed to 'DEG secreted' as suggested.

Line 449: The authors state, 'This was consistent for the whole set of analysed putative effectors.'but do not show the data. Is it available in a repository?

We have now provided a list of all the experiments carried out on the wider pool of 32 MEP effectors and provided all the results in Supplemental Data Set 8. This lists all of the mutants generated and reporter lines made. We have modifed text to make this completely clear.

Fig 6B: the GFP image here is very grainy compared to other images such as 6D, was the fluorescence much less intense? If so please provide the reader with some explanatory information (such as the microscope settings required to produce the image).

The resolution was not the same in the images and all have now been reproduced at the same resolution to deal with this difference. We have, specifically, provided new images for Fig 6B with the same format setting as all other panels in Figure 6.

Fig 8B: given that Mep1-GFPNLS has a signal peptide, how do the authors explain its retention in the fungal appressorium and localisation in the nucleoplasm and nucleolus? Surely the default secretory pathway operates in appressoria? How/why does the effector fusion exit the ER?

The reviewer is correct that, given that Mep1 contains a signal peptide, it should be channeled into the secretory pathway. However, we also know that Mep1 is very highly expressed in appressoria under its native promoter and therefore we assume that a proportion of the protein is able to enter they cytoplasm and therefore because of the

added nuclear localisation signal (NLS), will enter the nucleus, as we observe. The critical observation, however, is that Mep1 is an apoplastic effector and that the NLS added cannot direct it to the rice cell nucleus. This provides evidence that Mep1 does not enter rice cells.

Line 585: rather than same 'targets' it might be more realistic to suggest targeting of the same pathways or complexes.

The sentence has now been rephrased as suggested.

Line 596 and 7: new and two are misspelt

We have corrected the sentence.

TPC2022-LSB-00671R1 2nd Editorial decision – *accept with minor revision* Dec. 21, 2022

We have received reviews of your manuscript entitled "The transcriptional landscape of plant infection by the rice blast fungus *Magnaporthe oryzae* reveals distinct families of temporally co-regulated and structurally conserved effectors." On the basis of the advice received, the board of reviewing editors would like to accept your manuscript for publication in The Plant Cell. This acceptance is contingent on revision based on the comments of our reviewers. In particular, please consider the following:

Reviewer 1 acknowledged that the efforts and new data collected during the revision have improved the manuscript. However, this reviewer felt that the fitness assays using mixed inoculation provided interesting data but there were questionable perspectives on such assays. Therefore, the conclusions from these assays should be toned down with full discussion of all these scenarios. The authors should explain such assays are different from quantitative assays so that the readers are fully aware of these differences.

TPC2022-LSB-00671R2 2nd Revision received Jan. 3, 2023

Reviewer comments and **author responses**:

Editor:

Reviewer 1 acknowledged that the efforts and new data collected during the revision have improved the manuscript. However, this reviewer felt that the fitness assays using mixed inoculation provided interesting data but there were questionable perspectives on such assays. Therefore, the conclusions from these assays should be toned down with full discussion of all these scenarios. The authors should explain such assays are different from quantitative assays so that the readers are fully aware of these differences.

We have responded to these comments by incorporating a section in the Discussion section (Lines 626-641) that describes the limitations in the relative fitness assay and the need for caution in the interpretation of the results.

Reviewer Comments

Does mixed inoculation lead to the formation of one lesion with multiple independent penetration events of the mutant and WT? My understanding is that we don't know about this. However, only a limited number of lesions are normally formed on leaves sprayed with thousands of M. oryzae conidia under laboratory conditions. I assume that only a small percentage of conidia are successful at penetration and lesion development. It is possible that, on leaves inoculated with mixed conidia, most of the time, WT and mep1 conidia penetrated and formed lesions independently (not co-penetration). For most of these lesions, there may be no direct contact or competition between WT and mep1 mutant. With the method described in this manuscript, any mutants with defects in conidiation on lesions will be tested positive to have a fitness defect in competition. Same concerns for any mutants with defects in invasive growth or lesion development and the mep1 mutant is reduced in virulence. Therefore, to this reviewer, this approach or method to assess 'fitness' of a mutant by mixed inoculation is questionable and may lead to data overinterpretation

We have responded to the comments by incorporating a section into the Discussion (Lines 626-641) which we feel address the concerns of the Reviewer.

To address the comments directly it is important to recognize that the relative fitness assay is operating at a population level. We now explicitly state this on line 504 of the Results. It is likely that most infections by mutant and wild type do result in separate lesions. The reviewer is right, there is probably no direct competition between mutant and wild type within the same plant cell. The competition is operating at the population level. If a mutant lacking an effector is less able to generate a lesion, or if the lesions that result are smaller, or produce less conidia, then over generations, the mutant will be less prevalent in the pathogen population and, ultimately, will become extinct. This is how the assay works and this is the fitness coefficient that is being measured.

> **The limitations of the assay are that conidiation from disease lesions is being used a proxy measure of the success of an infection. This clearly has limitations, as identified by the Reviewer and needs to be understood, so we have now made this as clear as we can in the discussion. We have also highlighted that the assay cannot reveal why an effector mutant is less virulent, or measure its rate of cell invasion or tissue colonisation. We highlight that other assays are necessary, but also that they are hard to carry out at scale. We absolutely agree with the reviewer that quantitative cell biological analysis of cell invasion is necessary to evaluate the function of an effector and also that measuring biomass in colonised leaves is important. However, when the differences observed are very small, as often reported for effector mutants, we think it is valuable to look at pathogen survival at a population level which may be more representative of the situation in the field and might help us understand why losing an individual effector– even from a large effector repertoire –might be detrimental to the pathogen. It is for this reason that we think that the relative fitness assay may have some utility. We have, however, toned down the conclusions and incorporated all of the concerns indicated.**

TPC2022-LSB-00671R2 3rd Editorial decision – *acceptance pending* **Jan. 9, 2023**

We are pleased to inform you that your paper entitled "The transcriptional landscape of plant infection by the rice blast fungus *Magnaporthe oryzae* reveals distinct families of temporally co-regulated and structurally conserved effectors" has been accepted for publication in The Plant Cell, pending a final editorial review by a science editor.

Final acceptance from Science Editor **Jan. 27, 2023**
